# A minimum catalytic unit for synthesis of InsP_6_ and 5-PP-InsP_5_ in Arabidopsis

**DOI:** 10.1042/BCJ20253161

**Published:** 2025-12-17

**Authors:** Hayley L. Whitfield, Colleen Sprigg, Andrew M. Riley, Barry V.L. Potter, Hui-Fen Kuo, Charles A. Brearley

**Affiliations:** 1 School of Biological Sciences, University of East Anglia, Norwich Research Park, Norwich NR4 7TJ, UK; 2 Medicinal Chemistry & Drug Discovery, Department of Pharmacology, University of Oxford, Oxford OX1 3QT, UK; 3 Agricultural Biotechnology Research Centre, Academia Sinica, Taipei 115, Taiwan

## Abstract

Inositol pyrophosphates, diphosphoinositol phosphates, are reported agents of phosphate homeostasis, disease resistance and hormone action in plants. Of the enzymes that have been shown to synthesize inositol pyrophophosphates, ITPK1 and VIH1/2 share the ATP-grasp fold – the latter also possesses a phosphatase domain. Among ATP-grasp inositol phosphate kinases, ITPK1 is particularly flexible – phosphorylating equatorial hydroxyls and equatorial phosphates on inositol phosphates. Herein, we show that combination of ITPK1 and IPK1 is sufficient to synthesize 5-PP-InsP_5_ from Ins3P and that ITPK1 is capable of converting Ins1P to Ins(1,3,4,5,6)P_5_. In defining a minimal catalytic unit for synthesis of both InsP_6_ and 5-PP-InsP_5_, we define the minimum enzymology of the ‘lipid-independent’ pathway of InsP_6_ synthesis from Ins3P and its intermediates. The pathway proceeds: Ins3P, Ins(3,4)P_2_, Ins(3,4,5)P_3_, Ins(3,4,5,6)P_4_, Ins(1,3,4,5,6)P_5_, Ins(1,2,3,4,5,6)P_6_, and therefrom to 5-PP-Ins(1,2,3,4,6)P_5_. Sentence removed

## Introduction

Plants, animals and yeast possess orthologs of D-*myo*-inositol 3-phosphate synthase (MIPS, also known as IPS or ISYN) that catalyses the cyclo-aldolization of D-glucose 6-phosphate to D-Ins3P (1). As such, inositol phosphate metabolism is one step removed from glycolysis. Dephosphorylation of Ins3P provides inositol that is incorporated into phosphatidylinositol, and therefrom to phosphatidylinositol phosphates, by phosphatidylinositol synthase. In *Saccharomyces cerevisiae*, phospholipase C, IPK2 (IPMK, ARG82) and IPK1 comprise a lipid-dependent pathway that synthesizes InsP_6_ from the phosphoinositide PtdIns(4,5)P_2_ via Ins(1,4,5)P_3_, Ins(1,3,4,5)P_4_/Ins(1,4,5,6)P_4_ and Ins(1,3,4,5,6)P_5_ intermediates (2). The same pathway was also described in *Schizosaccharomyces pombe* (3). By contrast, a lipid-independent pathway of InsP_6_ synthesis starting with inositol was described in the protist *Dictyostelium discoideum* (4) and plants, duckweed (5–7). The extent to which the two canonical pathways intersect is poorly defined. Nevertheless, a single point of consensus across varied taxa is that phosphorylation of the axial 2-OH of Ins(1,3,4,5,6)P_5_ is the predominant final step of InsP_6_ synthesis in Dictyostelium, fungi, plants and animals, whether by nuclear or cytosolic activities (2,4,8), reviewed (9). It is unknown, however, whether Ins(1,3,4,5,6)P_5_ is the only intermediate shared between lipid-dependent and lipid-independent pathways (10).

Yeast lack the inositol tris/tetrakisphosphate (ITPK) family of ATP-grasp kinases present in plants and animals, while both yeast and plants lack IP3 3-K that phosphorylates the 3-hydroxyl of Ins(1,4,5)P_3_ which is generated following cell-surface receptor activation in animals (9). Though not widely appreciated, a route of Ins(1,3,4,5,6)P_5_ synthesis from Ins(3,4,6)P_3_ was described in avian erythrocytes (11), in which InsP_6_ is a minor component (12). Moreover, angiotensin-stimulated generation of Ins(3,4,5,6)P_4_ and subsequent conversion to Ins(1,3,4,5,6)P_5_ (13,14) made it likely that enzymes other than PLC, IPK2 and IPK1 participate in InsP_6_ synthesis in animals (reviewed 10). Similar conclusions were reached in early genetic studies of inositol phosphate synthesis in plants (15,16). This proposition was, however, only recently demonstrated formally in animals by ^3^H-inositol labeling of ITPK1 knock-outs in human HT-29 and HCT116 cells, albeit without identification of isomers (17,18). Notwithstanding all the above, the intermediates by which inositol monophosphates are converted by known enzymes to InsP_6_ remains an unanswered question; one that offers potential molecular explanation of diverse phenomena.

In plants, the ITPK family has diversified in structure, catalytic activity and physiological function (19–22). Disruption of family members reduces InsP_6_ accumulation in species from taxa that include maize (15), rice (23,24), oilseed rape (20,25) and Arabidopsis (26,27). Because substitution of the inositol ring underpins the specificity of inositol phosphate function, we have started by investigation of the catalytic repertoire of *At*ITPK1 and enquire whether disruption of ITPK1 has broader effects on inositol metabolism. Herein we describe intermediates of phosphorylation of Ins3P to Ins(1,3,4,5,6)P_5_ by *At*ITPK1, production of InsP_6_ therefrom, by *At*IPK1, and further phosphorylation to 5-PP-InsP_5_ by *At*ITPK1. We show, as in other species (23,24), that disruption of ITPK1 increases inositol levels in Arabidopsis.

## Results

### Successive phosphorylation of Ins3P by AtITPK1

**Figure 1:**
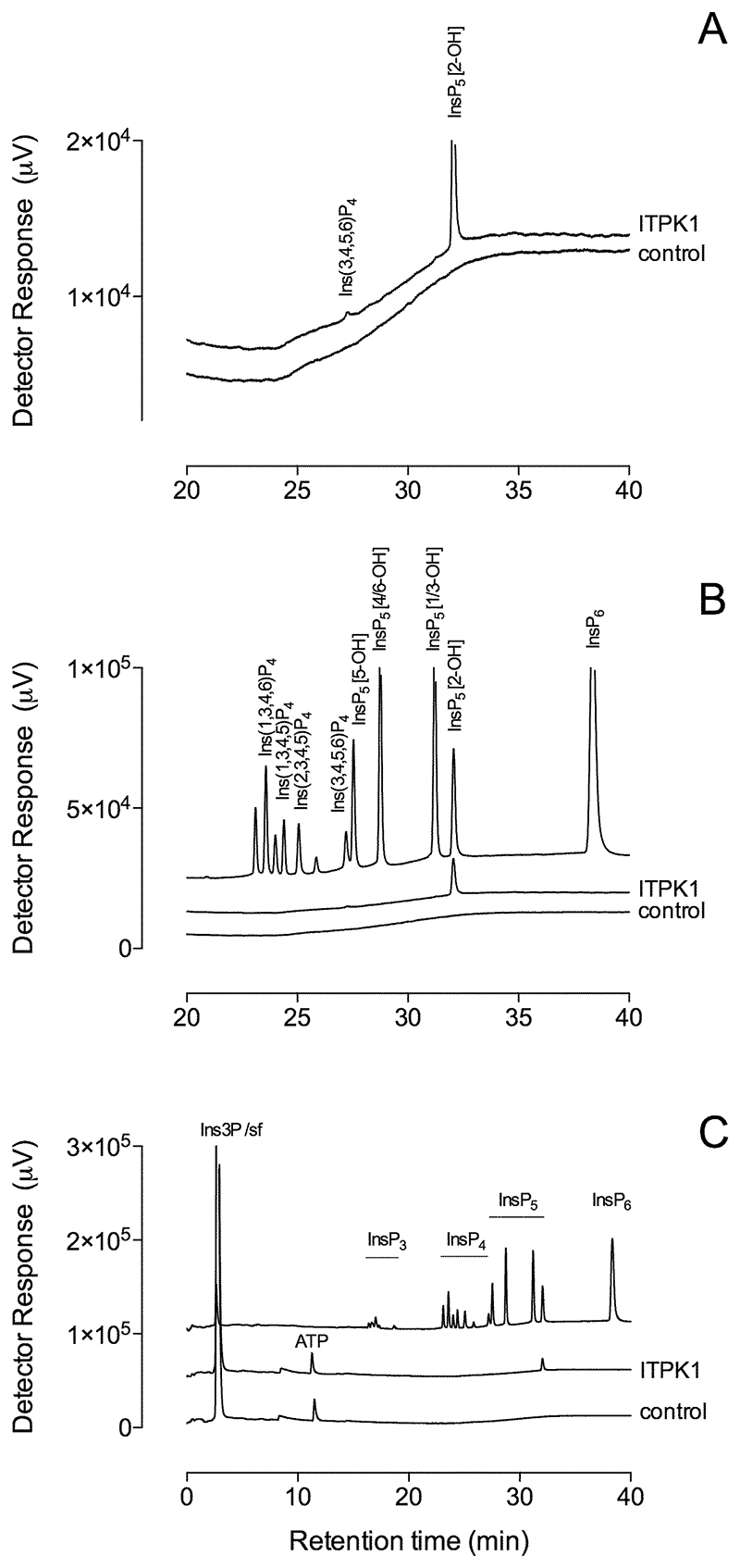
ITPK1 converts Ins3P to Ins(1,3,4,5,6)P_5_. Products of reaction of Ins3P with ITPK1 and ATP were resolved by HPLC on CarboPac PA200 eluted with methanesulfonic acid. Inositol phosphates were detected with ferric ion. **A**, an exploded view of the elution of InsP_4_ and InsP_5_ peaks in reaction products. **B**, an exploded view of the elution of InsP_4_ and InsP_5_ isomers, some of especial relevance to the ensuing discussion: Ins(1,3,4,6)P_4_, Ins(1,3,4,5)P_4_ and Ins(1,3,4,5,6)P_5_ (InsP_5_ [2-OH]), is shown beside products of phosphorylation of Ins3P. **C**, the position of elution of broad classes of inositol phosphate, viz., InsP_3_, InsP_4_ and InsP_5_ and InsP_6_, and ATP is shown. Ins3P elutes with the solvent front (sf). This chromatography has been repeated on more than thirty occasions, with Ins(1,3,4,5,6)P_5_ the single InsP_5_ product. The single *x*-axis label of panel C applies to panels A and B.

Previous characterization of *At*ITPK1 and *St*ITPK1 (28,29) shows that plant ITPK1 has robust Ins(3,4,5,6)P_4_ 1-hydroxykinase activity, 2 to 3 orders of magnitude greater than its InsP_6_ or PP-InsP_5_ phosphokinase activity (29–32). Here, *At*ITPK1 converted Ins3P to the *meso*-compound Ins(1,3,4,5,6)P_5_, along with a minor peak of intermediate level of phosphorylation that co-elutes with Ins(3,4,5,6)P_4_ (Figure 1). Even though Ins(1,4,5,6)P_4_ is not separable from its enantiomer Ins(3,4,5,6)P_4_ on non-chiral chromatography, the simplest explanation of InsP_4_ production is that the 3-phosphate is retained. The alternative possibility that the 3-phosphate is removed and replaced with a phosphate in the 1-position, while more involved, would allow for conversion to Ins(1,4,5,6)P_4_ that coelutes with Ins(3,4,5,6)P_4_.

**Figure 2:**
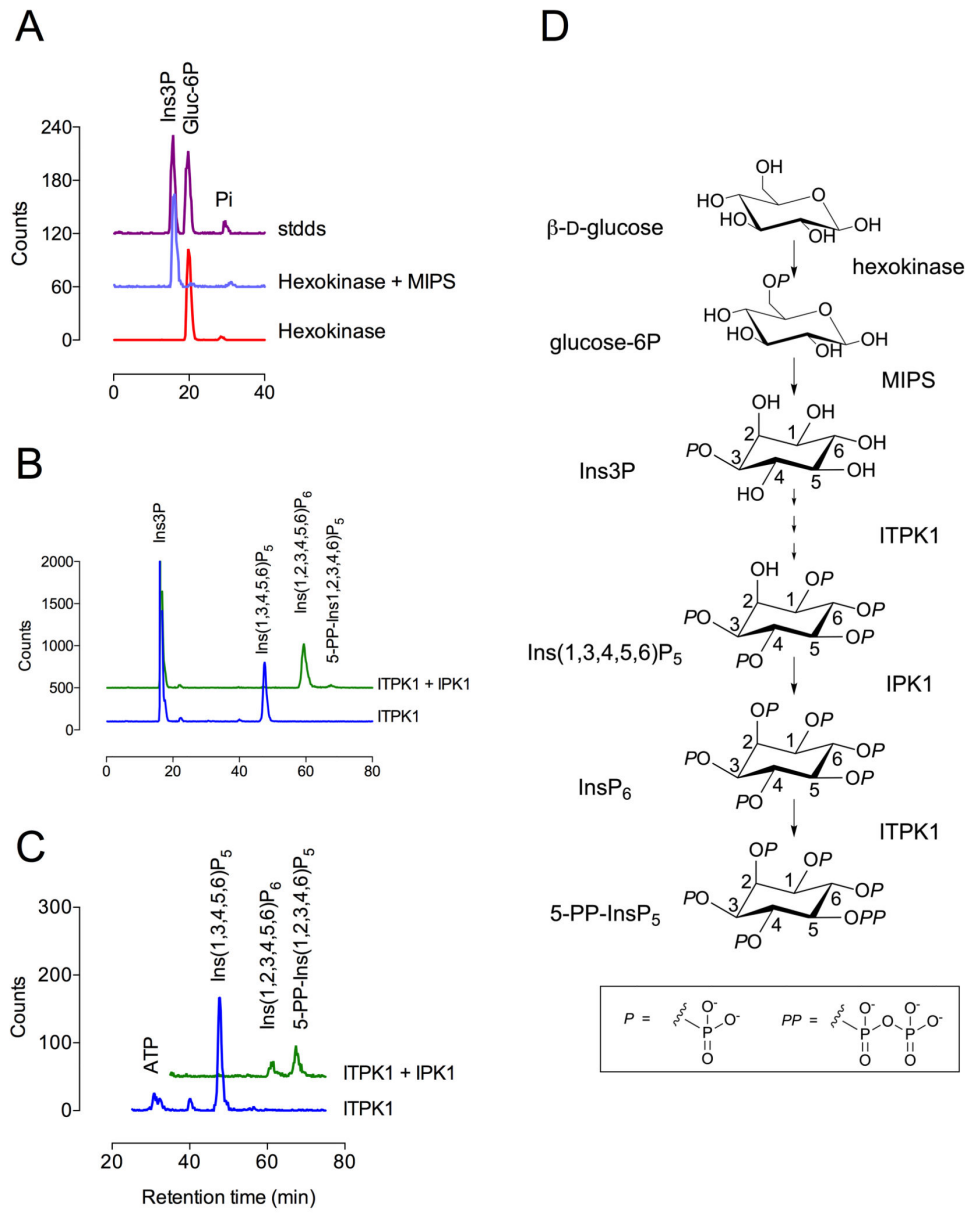
A four-enzyme conversion of glucose to 5-PP-InsP_5_. **A**, HPLC of conversion of D-glucose to [^32^P]-D-glucose 6-phosphate with hexokinase and [γ-^32^P]-ATP (red trace), and therefrom to [^32^P]-Ins3P with MIPS (blue trace), with HPLC of a mixed sample (purple trace). Products resolved on Partisphere SAX eluted with 40mM [NH_4_]_2_HPO_4_. **B**, HPLC of conversion of [^32^P]-Ins3P to Ins(1,[^32^P]3,4,5,6)P_5_ with ITPK1 (blue trace) and, by inclusion of IPK1, to 5-PP-Ins(1,2,[^32^P],3,4,6)P_5_ (green trace), Products resolved on Partisphere SAX eluted with a gradient of [NH_4_]_2_HPO_4_. **C**, HPLC of conversion of unlabeled Ins(1,4,5,6)P_4_ to Ins(1,[^32^P]3,4,5,6)P_5_ with ITPK1 and [γ-^32^P]-ATP (blue trace), and therefrom to [^32^P]-InsP_6_ and, [^32^P]-5-PP-Ins(1,2,3,4,6)P_5_ by addition of IPK1 (green trace). HPLC on Partisphere SAX eluted as B. In B and C, ^32^P label added into the 3-position of Ins(1,3,4,5,6)P_5_ (from [γ-^32^P]-ATP) is retained in InsP_6_ and 5-PP-InsP_5_ products. **D**, summary of synthesis scheme. The single *x*-axis label of panel C applies to panels A and B.

To prove retention of the 3-phosphate and remove any ambiguity surrounding the regiomeric character of 1- vs 3- phosphates in intermediates, [^32^P]-Ins3P was synthesized. First, [^32^P]-glucose 6-phosphate was synthesized from glucose and [γ-^32^P]-ATP with yeast hexokinase. Subsequently, by driving the reaction with excess glucose, and an ATP-regenerating system > 95% of the ATP was converted (Figure 2A). Using thermostable MIPS from *Archeoglobus fulgidus*, [^32^P]-glucose 6-phosphate, was converted to [^32^P]-Ins3P (Figure 2A).

**Figure 3:**
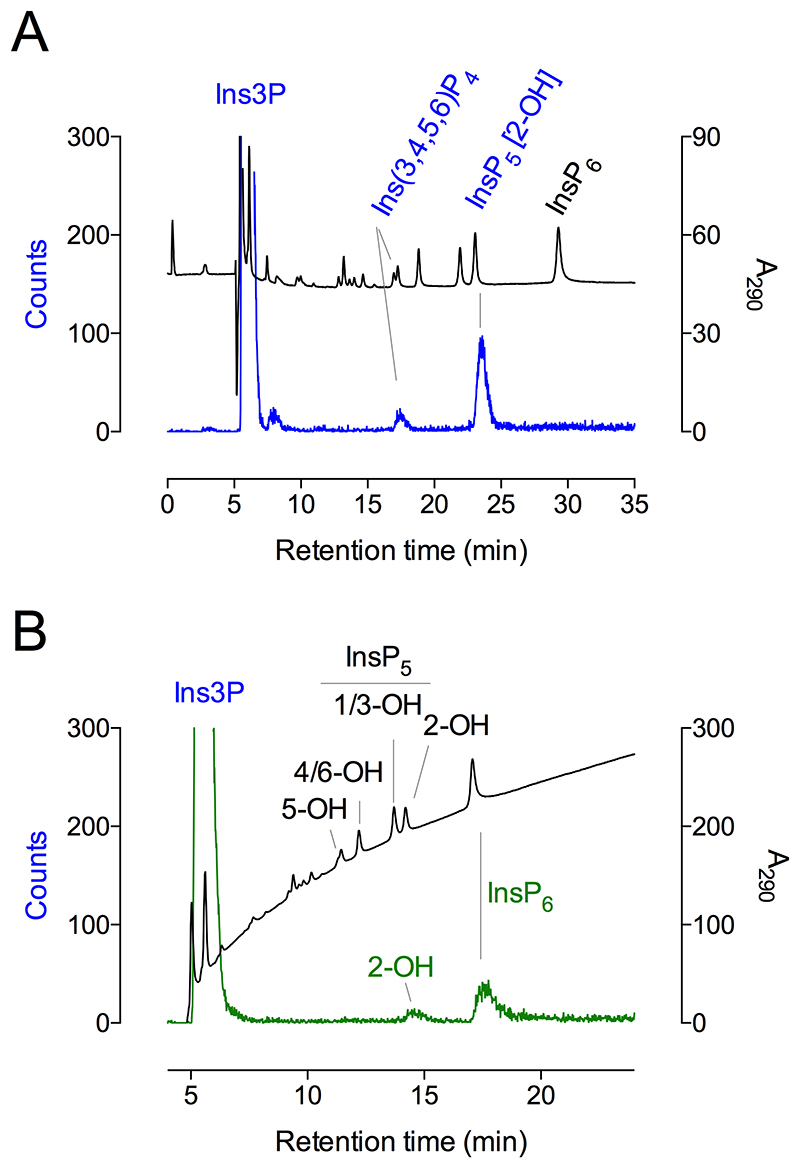
Retention of ^32^P label during conversion of Ins3P to InsP_6_ via Ins(3,4,5,6)P_4_ and Ins(1,3,4,5,6)P_5_. **A**, HPLC on CarboPac PA200 eluted with methanesulfonic acid of products of assay of [^32^P]-Ins3P with ITPK1 (blue trace). **B**, products from assay with ITPK1 and IPK1 (green trace) were eluted with HCl. Elution of inositol phosphate standards spiked into the samples and detected with ferric ion are shown (black traces).

Addition of ITPK1 and an ATP-regenerating system allowed phosphorylation of [^32^P]Ins3P to ^32^P-labeled InsP_4_ and InsP_5_ products, resolved on different HPLC phases (Figure 2B, Figure 3A,B). The precise elution of [^32^P]-InsP_4_ with Ins(1,4,5,6)P_4_/Ins(3,4,5,6)P_4_ and retention of the ^32^P-labeling unambiguously identifies Ins(3,4,5,6)P_4_ as an intermediate in the conversion of Ins3P to Ins(1,3,4,5,6)P_5_. Previously, the products of phosphorylation of Ins(3,4,5,6)P_4_ by the ITPK1/IPK1 couple were shown to be Ins(1,3,4,5,6)P_5_ (InsP_5_ [2-OH]), InsP_6_ and 5-PP-InsP_5_ (5-InsP_7_) (28). Thus, from Ins(3,4,5,6)P_4_ all successive phosphorylations generate achiral (*meso*-) products. In the current study, inclusion of IPK1 allowed retention of the ^32^P-labelled 3-phosphate (of Ins3P) in InsP_6_ (Figure 2B,D, Figure 3A,B) and 5-PP-InsP_5_ (Figure 2B,D). Ins(1,4,5,6)P_4_ is a weaker substrate of ITPK1 than its enantiomer Ins(3,4,5,6)P_4_ (26,27), its use here confirmed that addition of a 3-phosphate is retained in the InsP_6_ and 5-PP-InsP_5_ products of the combined action of ITPK1 and IPK1 (Figure 2C).

### Identification of intermediates in the conversion of Ins3P to Ins(1,3,4,5,6)P_5_

Except for generation of a peak with the mobility of In(3,4,5,6)P_4_, radiolabeled peaks intermediate in phosphorylation between InsP and InsP_4_ were trivial components and not reliably observed. Similar conclusions can be drawn for human ITPK1 (18). Chromatography on CarboPac PA200 similarly failed to reveal peaks with chromatographic properties of InsP_2_ and InsP_3_ (Figure 1).

Taking a candidate intermediate approach to phosphorylation of Ins3P, phosphorylation of the axial 2-hydroxyl can be discounted because ITPK1 does not add a 2- phosphate to a wide range of tested substrates (28,29) and because addition of IPK1 is required to phosphorylate the ‘vacant’ 2-hydroxyl of the [^32^P]InsP_5_ product later in the sequence (Figure 2C, Figure 3B). The only enzyme yet shown to possess this activity across plant, metazoan and fungal taxa is IPK1 which has a different structural fold. Similarly, Ins(1,3)P_2_ can be discounted as a product because the 1-phosphate is added later, in the conversion of Ins(3,4,5,6)P_4_ to Ins(1,3,4,5,6)P_5_ (Figure 2B,D, Figure 3A). This leaves the three ‘undetected’ possible InsP_2_ products of phosphorylation of Ins3P as Ins(3,4)P_2_, Ins(3,5)P_2_ and/or Ins(3,6)P_2_. By similar principles, the InsP_3_ product could be Ins(3,4,5)P_3_ or Ins(3,4,6)P_3_, but not Ins(4,5,6)P_3_.

**Figure 4:**
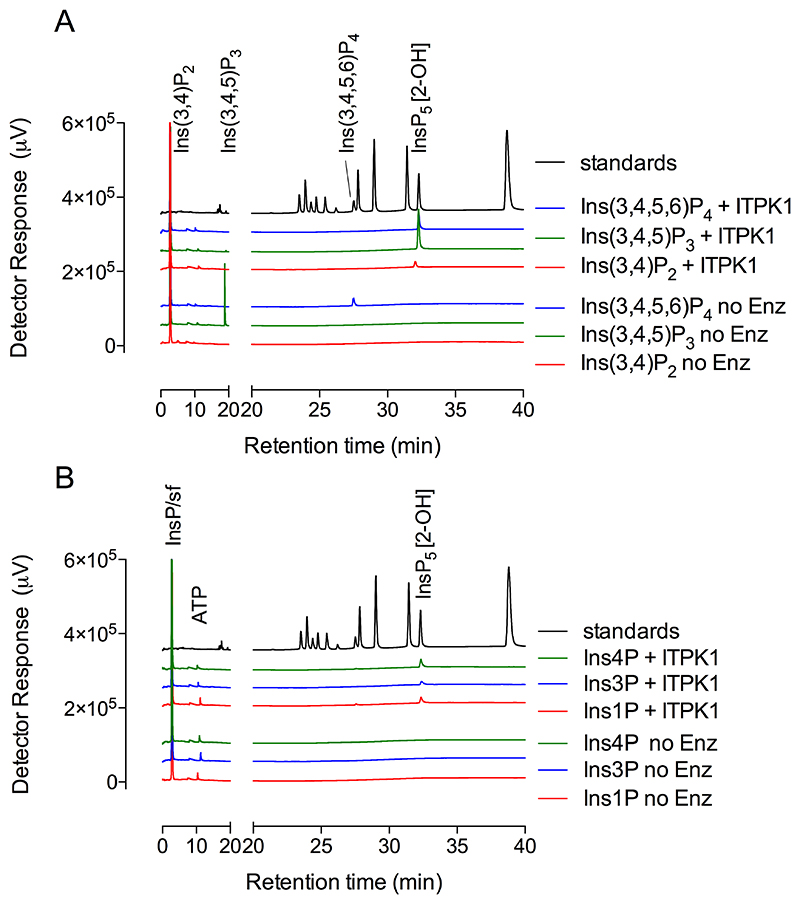
ITPK1 converts 3-phosphorylated inositol phosphates to Ins(1,3,4,5,6)P_5_. **A**, products of 60-minute assay of ITPK1, 200 μM ATP and 100 μM inositol phosphate were resolved by CarboPac PA200 HPLC eluted with methanesulfonic acid, with detection with ferric ion: Ins(3,4)P_2_ (red trace); Ins(3,4,5)P_3_ (green trace); Ins(3,4,5,6)P_4_ (blue trace). Control assays incubated for 60 minutes without enzyme are shown, Ins(3,4)P_2_ (red trace); Ins(3,4,5)P_3_ (green trace); Ins(3,4,5,6)P_4_ (blue trace). **B**, products of 60-minute assay of ITPK, 500 μM ATP and 500 μM inositol phosphate: Ins1P (red trace); Ins3P (blue trace); Ins4P (green trace). Control assays incubated for 60 minutes without enzyme are shown, Ins1P (red trace); Ins3P (blue trace); Ins4P (green trace). Inositol monophosphate substrates elute in the solvent front, sf. In A and B, elution of inositol phosphate standards are shown (black trace).

Both Ins(3,4)P_2_ and Ins(3,4,5)P_3_ were converted to Ins(1,3,4,5,6)P_5_ (Figure 4A), while Ins(3,4,6)P_3_ was converted to peaks with the chromatographic properties of Ins(1,3,4,6)P_4_ and Ins(1,3,4,5)P_4_ (Table 1). The absence of commercially available Ins(3,5)P_2_ and Ins(3,6)P_2_ precluded their analysis, but the efficient conversion of Ins(3,4,5)P_3_ is consistent with Ins(3,4)P_2_ as precursor.

In summary, *At*ITPK1 is shown to convert Ins3P to Ins(1,3,4,5,6)P_5_ via Ins(3,4)P_2_, Ins(3,4,5)P_3_ and Ins(3,4,5,6)P_4_ intermediates (Figure 4A,B).

### Kinetics of Ins3P phosphorylation

**Figure 5:**
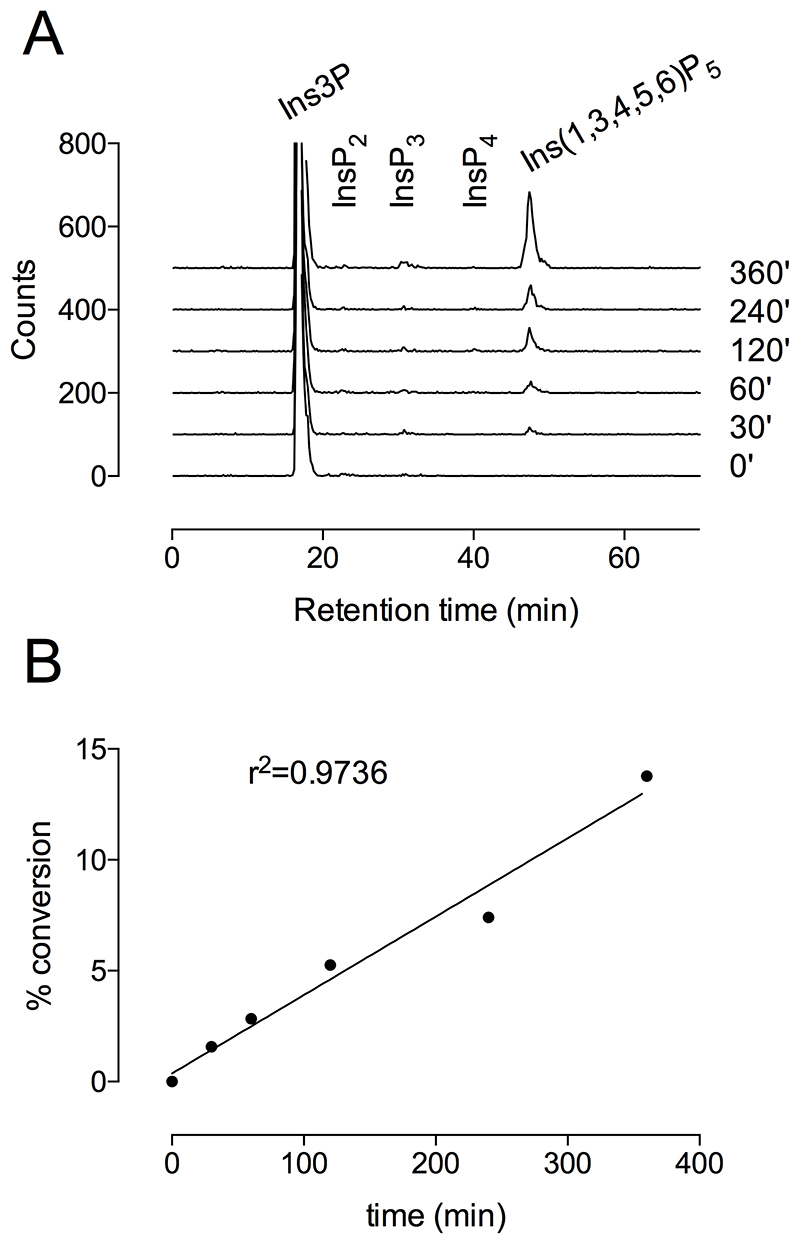
Kinetics of [^32^P]Ins3P phosphorylation to Ins(1,3,4,5,6)P_5_. **A**, products of reactions were analysed by HPLC on Partisphere SAX. **B**, percentage conversion to Ins(1,3,4,5,6)P_5_.

To determine kinetics of phosphorylation of Ins3P, we used [^32^P]-Ins3P as substrate. The appearance of ^32^P-labeled products was followed over a time-course. The approach allows us to conclude that the reaction was not substrate limited, with less than 14 % conversion of starting material at the final time point (Figure 5A,B). The standard error of the linear regression as calculated by GraphPad Prism v.6 yielded a rate constant of 19.42±0.800 nmol min^-1^ mg^-1^ (0.70±0.029 min^-1)^. This value is similar to that with InsP_6_ substrate but is a small fraction of that obtained with Ins(3,4,5,6)P_4_ (28). At 6 h of reaction, a small peak of a putative [^32^P]InsP_3_ was evident on Partisphere SAX (Figure 5A).

### Phosphorylation of Ins1P

In vitro, human ITPK1 phosphorylates Ins1P, as it does Ins3P, via unknown intermediates to unidentified InsP_5_ product(s) (18). *At*ITPK1 was tested for phosphorylation of Ins1P, the enantiomer of Ins3P. Ins1P was converted to Ins(1,3,4,5,6)P_5_ (Table 1, Figure 4B). Similarly, Ins(1,3)P_2_, Ins(1,5)P_2_, and Ins(1,3,5,6)P_4_ were also converted to Ins(1,3,4,5,6)P_5_ (Table 1). Close inspection of our previous study (Figure 3D of (28)) revealed the production of a very small peak of Ins(1,3,4,5,6)P_5_ from Ins(1,3,5,6)P_4_, albeit trivial compared to that generated from Ins(3,4,5,6)P_4_. Here, various isomers of InsP_2_ and InsP_3_ were also tested as potential intermediates. Among these, Ins(1,4)P_2_, Ins(1,3,4)P_3_, Ins(1,3,5)P_3_, Ins(1,4,5)P_3_ and Ins(3,4,6)P_3_ were converted to Ins(1,3,4,6)P_4_ and/or Ins(1,3,4,5)P_4_ but not to Ins(1,4,5,6)P_4_, Ins(3,4,5,6)P_4_ or Ins(1,3,4,5,6)P_5_ (Table 1). Ins(1,3,4,5)P_4_ was interconverted (isomerized, (33–35)) to Ins(1,3,4,6)P_4_ without further phosphorylation (Table 1). In a previous study with assays at higher substrate concentration, we did not observe product from Ins(1,3,4,5)P_4_ or from Ins(1,3,4,6)P_4_ (28).

**Figure 6:**
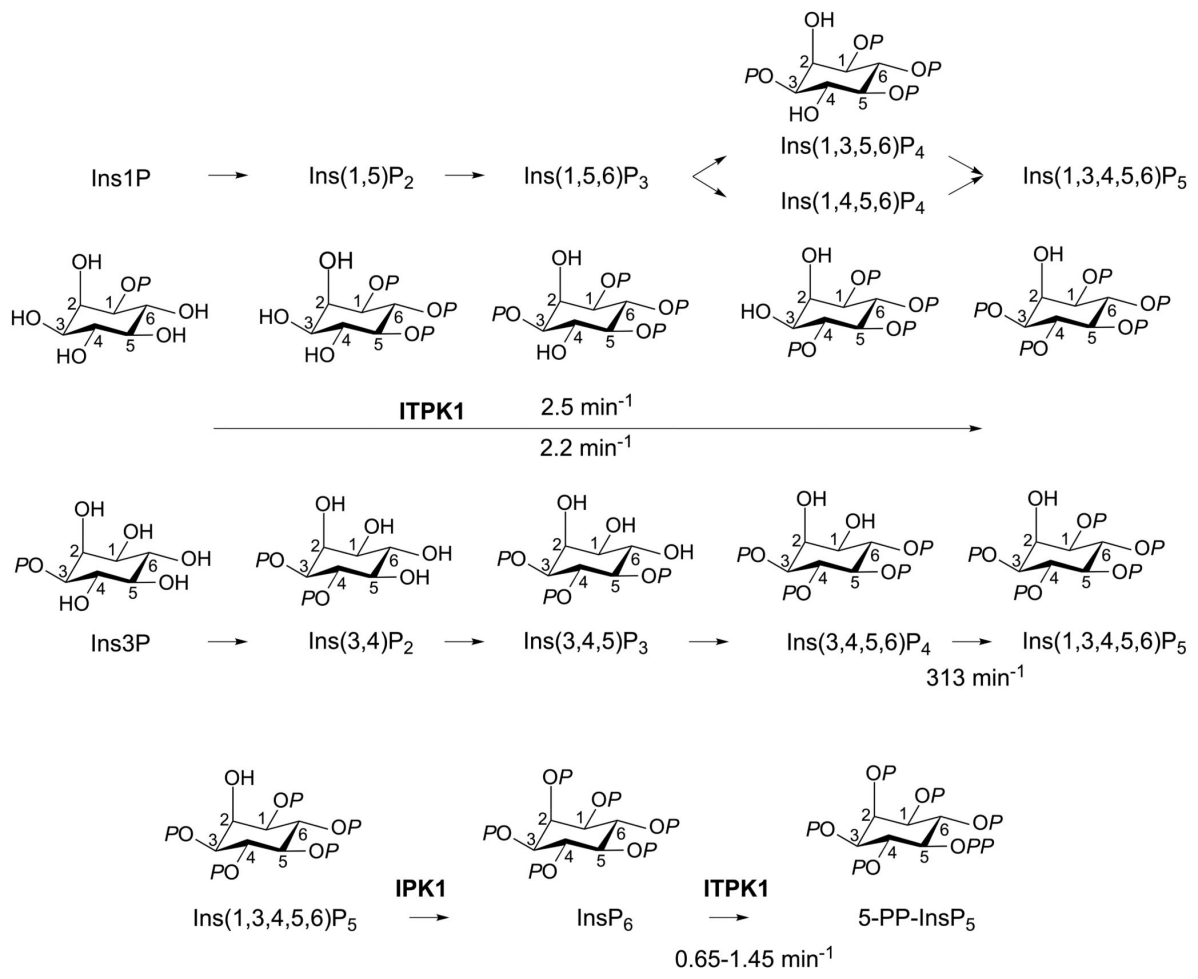
The contribution of ITPK1 to inositol phosphate synthesis. Rate constants for individual and aggregate steps are indicated. For Ins1P, the involvement of Ins(1,3,6)P_3_ and Ins(1,5,6)P_3_ are speculations based on demonstrated conversion of Ins(1,3)P_2_ and Ins(1,5)P_2_, and of Ins(1,3,5,6)P_4_ and Ins(1,4,5,6)P_4_, to Ins(1,3,4,5,6)P_4_ (Table 1 and text).

Collectively, these data suggest that all potential flux is channeled to Ins(1,3,4,5,6)P_5_ via Ins(1,3,5,6)P_4_ and/or Ins(1,4,5,6)P_4_ for Ins1P, and via Ins(3,4,5,6)P_4_ for Ins3P (Figure 6). Without Ins(1,6)P_2_ or Ins(1,3,6)P_3_, we were unable to test these as potential intermediates for phosphorylation of Ins1P but we speculate that both Ins(1,3,6)P_3_ and Ins(1,5,6)P_3_ are preferred intermediates in the conversion of Ins1P to Ins(1,3,4,5,6)P_5_.

ITPKs have been most intensively studied in relation to InsP_4_ and InsP_5_ turnover (13,14,40–42). To address how fluxes from ‘lower’ precursors, i.e., inositol monophosphates (10,18) might feed into Ins(1,3,4,5,6)P_5_ synthesis, we also considered Ins4P, even though this monophosphate is considered to be a product of Ins(1,4,5)P_3_ turnover in animal cells (9). Indeed, Ins4P was also converted to Ins(1,3,4,5,6)P_5_ (Table 1, Figure 4B). Given the inability of ITPK1 to convert Ins(1,4)P_2_ to Ins(1,4,5,6)P_4_ or Ins(1,3,4,5,6)P_5_, the absence of 2-hydroxykinase activity, the phosphorylation of Ins(3,4)P_2_ to Ins(1,3,4,5,6)P_5_, the conversion of Ins(4,5)P_2_ to Ins(1,3,4,5,6)P_5_ and of Ins(3,4,6)P_3_ only as far as Ins(1,3,4,6)P_4_ and Ins(1,3,4,5)P_4_ (Table 1), we speculate that Ins4P phosphorylation proceeds via Ins(3,4)P_2_ and/or Ins(4,5)P_2_, Ins(3,4,5)P_3_, and/or Ins(3,4,5,6)P_4_ to Ins(1,3,4,5,6)P_5_. Without Ins(4,6)P_2_, we were unable to test its potential contribution to Ins(1,3,4,5,6)P_5_ synthesis, but we note that neither Ins(1,4,6)P_3_ nor Ins(3,4,6)P_3_ yielded InsP_5_ product (Table 1).

In considering experiments such as these, we comment that where more than one product is generated at a particular level of intermediary phosphorylation - the intermediate most prominent is most likely the weaker substrate (i.e., the one left behind in the conversion of the better substrate to a ‘higher’ product). Indeed, in the scenario of ITPK1 action against Ins(1,4,5,6)P_4_ and Ins(3,4,5,6)P_4_, the former is the weakest substrate (28,29). It is possible that Ins(1,3,5,6)P_4_ is a better substrate than Ins(1,4,5,6)P_4_ and so when monophosphates are converted ‘all the way’ to Ins(1,3,4,5,6)P_5_, the only InsP_4_ peaks observed have the mobility of the Ins(1,4,5,6)P_4_/Ins(3,4,5,6)P_4_ enantiomeric pair.

### Comparison of Ins1P and Ins3P phosphorylation

**Figure 7:**
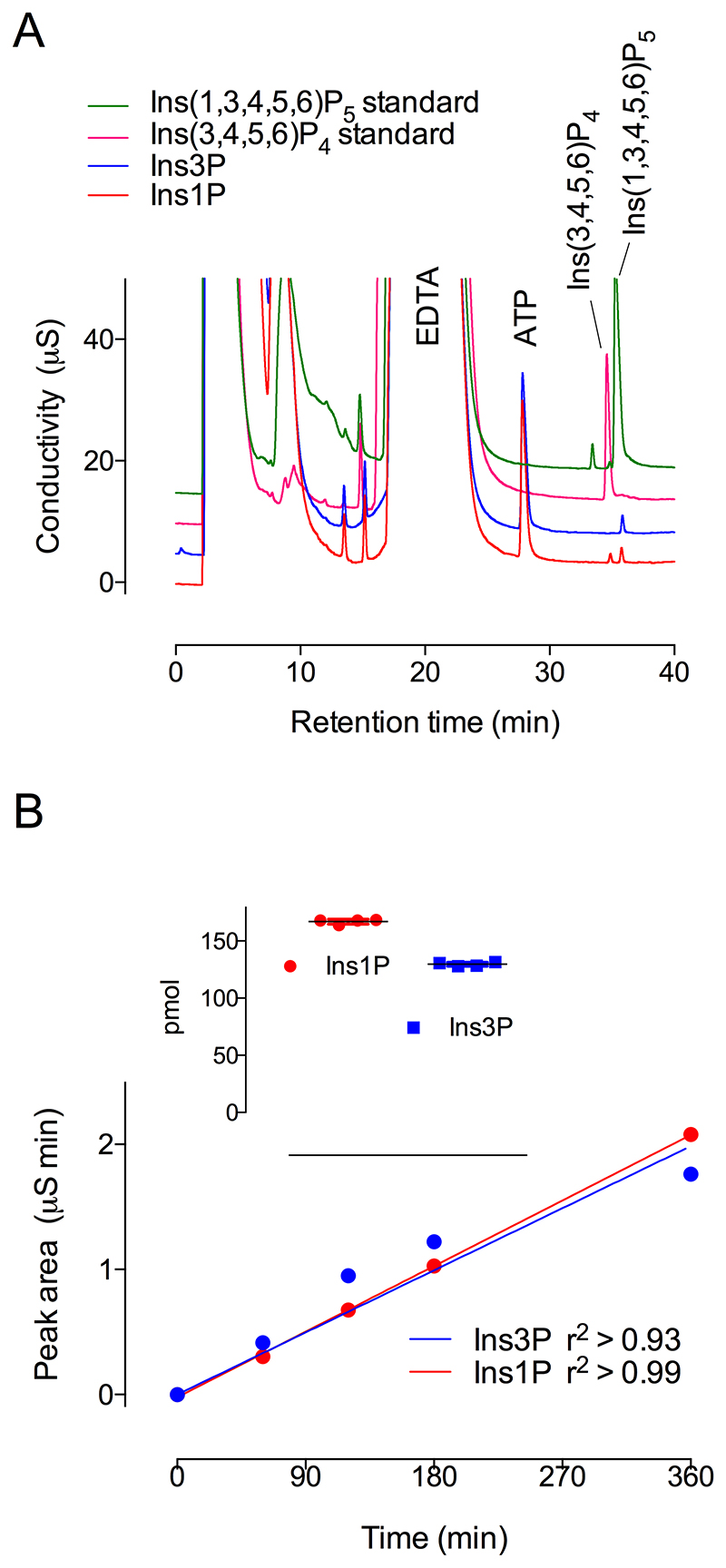
Suppressed ion-conductivity analysis of phosphorylation of Ins1P and Ins3P by ITPK1. Assays stopped by the addition of NaF, EDTA, pH 10, were eluted on a Dionex AS11 column. **A**, products generated from Ins1P (red trace) and Ins3P (blue trace) share retention time with Ins(3,4,5,6)P_4_ (cerise trace) and/or Ins(1,3,4,5,6)P_5_ (green trace) standards. The positions of elution of ATP and EDTA are shown. The chromatography shown in the figure, separation of Ins3P (or enantiomer Ins1P) from Ins(3,4,5,6)P_4_ (or enantiomer Ins(1,4,5,6)P_4_) and Ins(1,3,4,5,6)P_5_ has been repeated > 10 times. **B**, linearity of reaction with Ins1P and Ins3P from a time-course experiment with single determinations. Inset, 4 replicate measurements of product formation (InsP_4_ & InsP_5_, summed) from an independent experiment with samples taken at 2 h of incubation.

Direct comparison of the extent of reaction against Ins1P and Ins3P was determined from the production of InsP_4_ and InsP_5_, resolved on an AS11 column as quantified by conductivity (Figure 7). Here again, phosphorylation of Ins1P generated peaks of InsP_4_, coeluting with the Ins(1,4,5,6)P_4_/Ins(3,4,5,6)P_4_ enantiomeric pair, and of Ins(1,3,4,5,6)P_5_ (Figure 7A). The same was observed with ferric-based detection on a CarboPac PA200 column (Figure S1), whereby the absence of accumulation of InsP_2_ or InsP_3_ intermediates was again confirmed. In contrast, phosphorylation of Ins3P generated a predominant peak of Ins(1,3,4,5,6)P_5_ (Figure 7A) (and Figure S1). Limiting product accumulation to less than 5% of the starting substrate, in the linear period (Figure 7B), the macro constants (for inositol monophosphate conversion to product(s) InsP_4_ and/or InsP_5_) were similar for Ins1P and Ins3P. They are similar to that reported for InsP_6_, but are 2-3 orders of magnitude smaller than the rate constant for Ins(3,4,5,6)P_4_ phosphorylation (Table 2). These values explain the limited accumulation of InsP_2_ and InsP_3_ intermediates. We posit that ITPK1 ‘gathers pace’ with each successive phosphorylation, possibly in a processive manner, as far as Ins(1,3,4,5,6)P_5_

### The metabolic effects of disruption of Itpk1 extend to inositol

**Figure 8:**
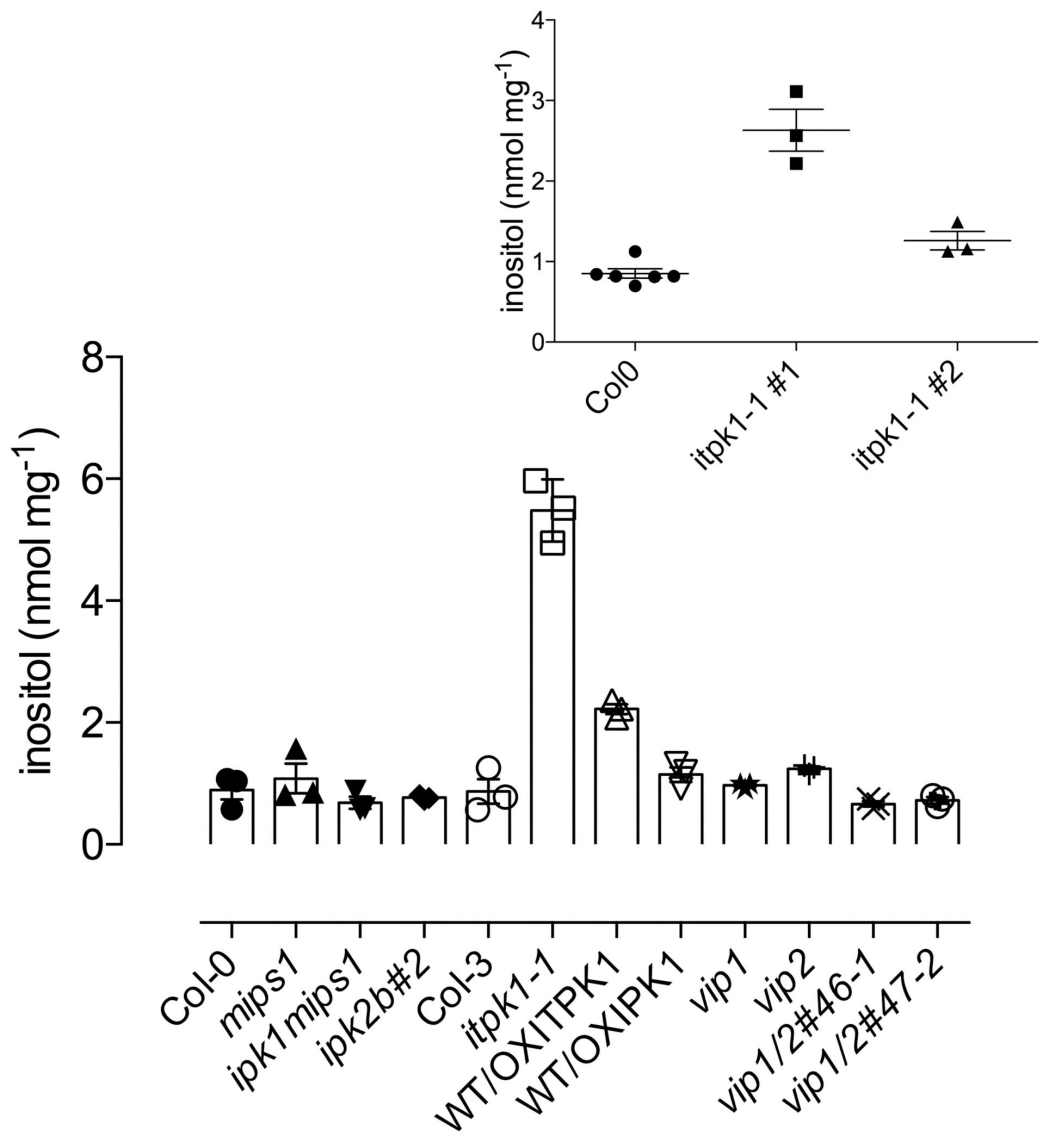
Inositol levels in seeds of various Arabidopsis genotypes. Inositol was measured by 2d-HPLC with pulsed amperometric detection, mean and se of n=3. The genotypes studied include Col0 (WT); *mips1*; a genetic cross between *ipk1* and *mips1, ipk1mips1*; ipk2ß; Col-3 (WT); *itpk1*; an overexpression line of ITPK1 in WT background, WT/OXITPK1; an overexpression line of IPK1 in WT background, WT/OXIPK1; and different alleles of *vip* mutants. Separate comparison of two *itpk1* lines (n=3) and Col-0 (WT, n=6) is shown in the inset. By two-tailed Student’s T-test, the means were significantly different at p < 0.05 between Col0 and the *itpk1* lines, both in the main panel and inset.

While varied studies have reported changes in profile of inositol phosphates in Arabidopsis mutants including *itpk1*, compared to wildtype, and effect on phosphate homeostasis (27,32,36–39), none have measured inositol. Indeed, while inositol is rarely described in studies of inositol phosphate or inositol pyrophosphate synthesis, it has been observed that disruption of ITPK orthologues not only reduces InsP_6_ but elevates inositol in rice (23,24). To test whether inhibition of inositol monophosphate conversion to Ins(1,3,4,5,6)P_5_ elevates inositol in Arabidopsis, inositol was measured in seeds of *itpk1* beside those of other mutants of inositol polyphosphate and pyrophosphate synthesis (Figure 8). They include *vip* (VIH) mutants widely reported to control phosphate homeostasis (reviewed, 39) and other mutants such as *ipk2ß* which reduce InsP_6_ levels in seeds and whose WT protein product, combined with IPK1, catalyzes InsP_6_ synthesis from Ins(1,4,5)P_3_ *in vitro* (38). Disruption of itpk1 elevated inositol up to 5-fold, while the other genotypes yielded inositol levels similar to wild-type (Figure 8).

## Discussion

Human ITPK1 synthesizes unidentified InsP_5_ isomer(s) from Ins1P and Ins3P and heterologous expression of itpk1, from archaea, plant, human and Dictyostelium in yeast *plc1*Δ causes the accumulation of InsP_6_ but does so by an unspecified series of intermediates (18). Deletion of ITPK1 in animal cell lines reduces InsP_6_ (17,18). While it was claimed for Ins3P substrate that the InsP_5_ product of heterologous expression in yeast of human ITPK1 cannot be Ins(1,3,4,5,6)P_5_ because expression of human ITPK1 in *plc1*Δ*arg82*Δ yeast did not restore InsP_6_ levels (from [^3^H]-inositol) (18), this argument was not validated by independent assignment of identity of the InsP_5_ specie(s) and rests also on limited analysis of InsP_3_ and InsP_4_ isomers in wild-type, mutant or ‘complemented’ yeast. Indeed, the assignment of identity in yeast and in mammalian cells is almost exclusively defined by radiolabeling on Partisphere SAX, which, as the authors show, is an incomplete description.

Here, we show that *At*ITPK1 synthesizes Ins(1,3,4,5,6)P_5_ from Ins3P, and that inclusion of *At*IPK1 enables accumulation of InsP_6_ and 5-PP-InsP_5_. Thus, ITPK1 and IPK1 constitute a minimum catalytic unit for PP-InsP synthesis in plants. That *At*ITPK1 generates Ins(3,4,5,6)P_4_ from Ins3P is consistent with previous characterizations of *At*ITPK1 and *St*ITPK1 activity towards Ins(3,4,5,6)P_4_ (28,29). While it is also claimed that mammalian ITPK1 is not responsible for synthesis of Ins(3,4,5,6)P_4_ from Ins(1,3,4,5,6)P_5_ (18), human ITPK1 has the Ins(3,4,5,6)P_4_ 1-kinase and Ins(1,3,4,5,6)P_5_ 1-phosphatase activities (40,41) that plant ITPK1 possesses (19,34,35). Metabolic studies also describe Ins(3,4,5,6)P4 1-kinase activities in plants (7,42,43).

It is perhaps remarkable that few have recognized the finely detailed analyses of inositol phosphate metabolism of avian erythrocytes from Stephens and coworkers, particularly (11), that reports Ins(3,4,6)P_3_ to be the precursor of Ins(3,4,5,6)P_4_ and Ins(3,4,5,6)P_4_ to be the principal precursor of Ins(1,3,4,5,6)P_5_. We speculate that these in vivo reactions are the manifestation of ITPK1 activity. The precedent also extends to mammals (13,14). Consequently, we propose that across metazoan taxa evolution has retained in ITPK1 activity against Ins3P and other ‘lower’ inositol phosphates, but with highest catalytic activity towards Ins(3,4,5,6)P_4_ (28,29).

The pathway of InsP_6_ synthesis from Ins3P described (Figure 6) differs at the InsP_3_ level, only, from that described in *Spirodela polyrhiza* (7): Ins(3,4,5)P_3_ in the former, Ins(3,4,6)P_3_ in the latter. Few groups have assigned stereoisomerism or enantiomerism to InsP_3_ species in plant tissues: there are 20 possibilities. Consequently, it is difficult to comment further. Also, the intermediates by which ITPK1 contributes to InsP_6_ synthesis in human cell lines are unknown (17,18), excepting the implications of the aforementioned work of Stephens (11), Balla (13,14) and Shears (40,41). We note, however, that the ITPK class is uniquely diversified in plants (19,20) and that Spirodela, a primitive vascular plant with vestigial roots occupies a clade that appears to have ‘returned to the water’ (44) and which shares an ITPK family structure of four orthologs with Arabidopsis.

Compared to wild-type, *itpk1* seedlings show elevations in ^3^H inositol-labeling of undefined InsP_3_ and InsP_4_ species (27,37) and elevation of labeling of Ins(1,4,5,6)P_4_ and/or Ins(3,4,5,6)P_4_, from ^32^P-orthophosphate (27). A reduction in ^3^H inositol-labeling of InsP_6_ was observed in the same study, but others (37) describe no such difference between wild-type and *itpk1*. This disparity possibly has its origins in the different ‘normalizations’ of the two studies. In the latter study uptake of inositol and labeling of inositol monophosphate is not accounted for, peaks are normalized to counts for peaks eluting thereafter, whereas in the former study peak areas are normalized to counts across the entire gradient. When comparing genotypes, normalization without considering inositol or all inositol phosphates risk the masking of genotype effect(s) on inositol phosphates and inositol pyrophosphates. Thus, for genotypes such as *mips* (43,45,46) in which phenotype extends to alterations of cellular inositol level (relative to wild-type) (45), and *itpk1* (this study), changes in specific activity of the inositol pool and subsequent labeling of inositol phosphates are treated differently by the two normalization approaches. Indeed, antisense inhibition of mips increased labeling of inositol phosphates from exogenous *myo*-[2-^3^H] inositol four to five - fold, an observation rationalized by increase in specific activity of the inositol pool (43).

**Figure 9:**
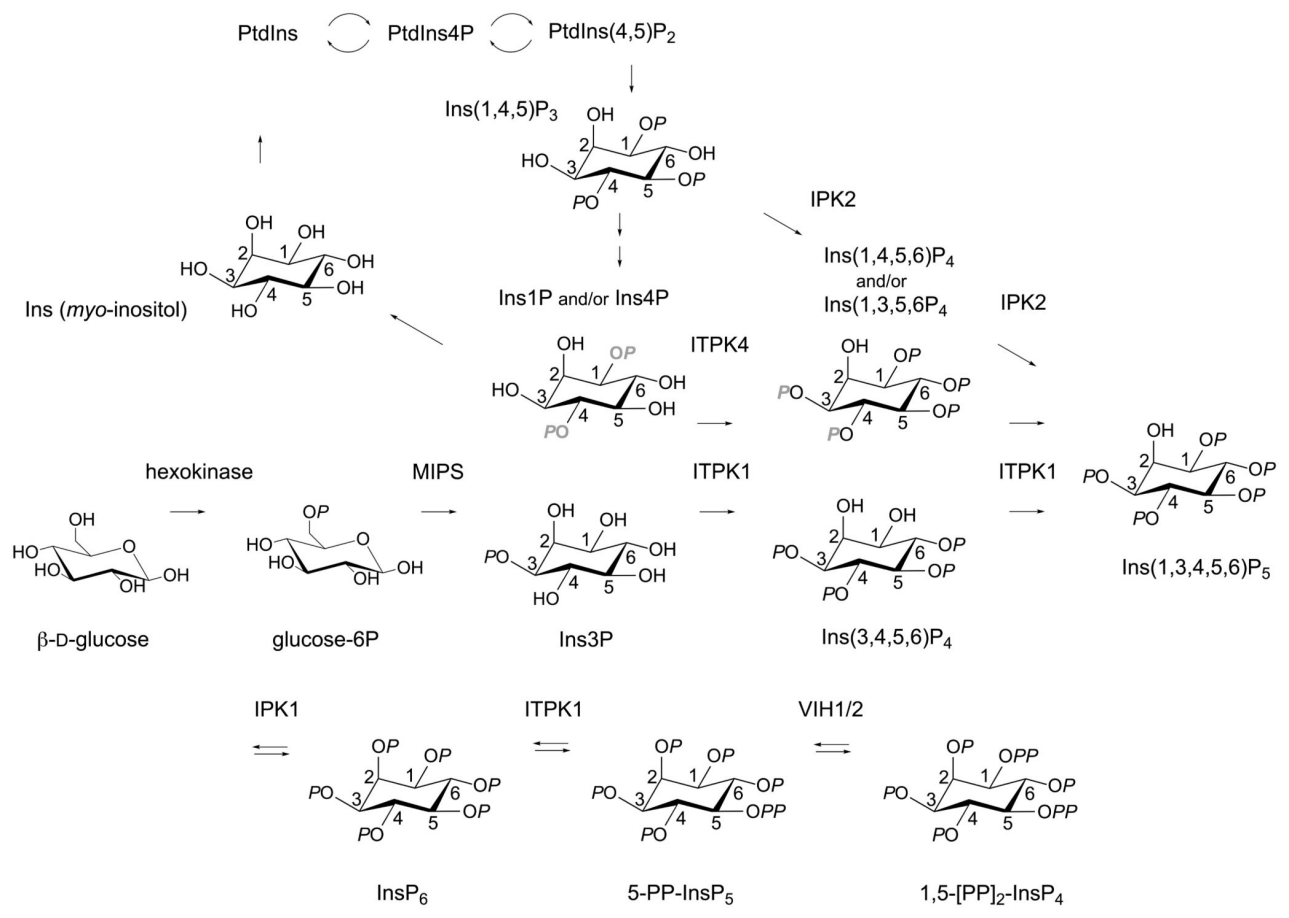
A revised metabolic network for InsP_6_ and PP-InsP synthesis in plants. ITPK1 integrates flux from the inositol monophosphate pool to Ins(1,3,4,5,6)P_5_ and from InsP_6_ to PP-InsPs.

This aside, ITPK1 is not the only ITPK contributor to InsP_6_ synthesis in Arabidopsis: *itpk4* mutants show much reduced levels, both of labeling from ^3^H inositol in seedlings (27), of InsP_6_ levels in plants (47) and of seed InsP_6_ levels (26,27). ITPK4 has reversed specificity for the Ins(1,4,5,6)P_4_/Ins(3,4,5,6)P_4_ enantiomeric pair, favouring Ins(1,4,5,6)P_4_ (21). Among the family, ITPK1 is also remarkably reversible and preferentially transfers the 1-phosphate of Ins(1,3,4,5,6)P_5_ to ADP, generating Ins(3,4,5,6)P_4_ (21,29). We posit that ITPK1 is the integrator of flux from Ins3P and potentially also from Ins1P and/or Ins4P to Ins(1,3,4,5,6)P_5_ and therefrom via IPK1 to InsP_6_ whether involving ITPK4 or other inositol phosphate kinases (Figure 9).

As in *Brassica napus* (20,25), maize (15) and rice (23,24), disruption of itpk orthologs reduces seed InsP_6_ (in Arabidopsis by c. 90% (26,27)). This is reminiscent of the effect in seeds and germinating seedlings of disruption of ipk1 (26,38). The authors (38) reported that the accumulating InsP_4_ peak comprises predominantly (80%) Ins(3,4,5,6)P_4_. Collectively, these data provide physiological context to the sequential action of ITPK1 and IPK1 in synthesis of InsP_6_ and of 5-PP-InsP_5_ (28,31), here from Ins3P. They also suggest that only a small part of InsP_6_ accumulation in Arabidopsis seeds is independent of ITPK1. The finding that Ins(1,3,5,6)P_4_ is a potential intermediate in conversion of Ins1P to Ins(1,3,4,5,6)P_5_ is itself an observation that could explain the accumulation of D/L-Ins(1,3,4,5)P_4_ in grain mutants, PLP1A, PLP2A and PLP3A, of barley (48): Ins(1,3,4,5)P_4_ is the enantiomer of Ins(1,3,5,6)P_4_, the enantiomers are not resolved chromatographically, nor are they distinguished by the NMR characterization (48).

Irrespective of the fine detail of ITPK1 involvement in InsP_6_ synthesis, disruption of itpk1 has pronounced effect on inositol – perhaps implying that the pleiotropic effects of ITPK1 on plant physiology, including those attributed to perturbation of inositol pyrophosphate signalling, may have their origins more broadly in inositol phosphate metabolism. An elevation of inositol was observed in itpk2 down-regulated rice RNAi (23,24). These data potentially explain the reductions in labeling of InsP_6_ from exogenous [^3^H]-inositol observed in vegetative tissues of *itpk1* (27), by simple dilution of label. An alternative mechanism for elevation of inositol levels in *itpk1* may lie in the equivalence of the roles of plant ITPK1 and mammalian IP6K1 for phosphorylation of InsP_6_ (28–32): Greenberg and coauthors (49) showed that disruption of IP6K1 (loss of 5-PP-InsP_5_) increases MIPS (ISYN, mINO1) transcription and elevates inositol levels.

## Methods

### Protein expression and purification

### *Af*IPS (MIPS)

*Archaeoglobus fulgidus* Inositol Phosphate Synthase (MIPS), pET23a: *Af*IPS (from Adolfo Saiardi), was expressed in Rosetta™ 2 (DE3)pLysS (Novagen). Cells were induced at 30°C with 0.5 M IPTG overnight in LB medium with ampicillin and chloramphenicol selection. Cells were resuspended in lysis buffer and lysed by French press in 50 mM NaH_2_PO_4_ pH 7.5, 300 mM NaCl, 20 mM imidazole, 1 % Triton-X-100 and protease inhibitor (Roche). It was determined that the protein did not interact with the NiNTA column since the NiNTA loading waste and wash A (50mM NaH_2_PO_4_ pH 7.5, 300mM NaCl, 20mM imidazole) contained the target protein, but wash B (50mM NaH_2_PO_4_ pH 7.5, 300mM NaCl, 250mM imidazole) did not (Figure S2). Lysate containing 2 mM DTT was heated to 80°C for 30 mins and centrifuged at 14,000x *g* for 15 minutes. Protein was concentrated to approximately 2 mg/ mL and stored at -80°C. Enzyme activity was verified using the assay described below.

*At*ITPK1 and *At*IPK1 were purified as described (28).

### Enzyme Assays

#### Three-step synthesis of [^32^P]-Ins3P

Conversion of glucose to 1D-*myo*-inositol-3-phosphate (Ins3P) was carried out as a three-step process. First, glucose was converted to glucose-6-phosphate (G6P) in 50 mM Tris-Ac pH 7.5 buffer containing 50 mM D-glucose, 1.5 mM ATP, 1850 kBq γ-^32^P ATP, 5 mM phosphocreatine, 5 mM MgCl_2_, 0.4 units yeast hexokinase and 6 units of creatine phosphokinase (from rabbit muscle, Merck Product # C3755) in 40 μL at 30°C for 2 h. To ensure all glucose was converted to G6P, further regeneration assay components were added (50 mM phosphocreatine, 15 units creatine phosphokinase, 1 mM DTT, 100 mM NaCl, 3 mM MgCl_2_) and incubated at room temperature for a further 3 h. Finally, to convert G6P to Ins3P, 1 mM NAD^+^, 1 mM ZnSO_4_ and 20 μg IPS enzyme was added to the previous reaction and incubated at 80°C for 4 h.

#### ITPK1 assays

Assays without radioactivity were carried out under regeneration conditions as described (28) in 10 μl reactions containing 20 mM HEPES pH 7.5, 1 mM MgCl_2_ at either 100 μM substrate, 200 μM ATP or 500 μM substrate, 500 μM ATP with 1.26 μM *At*ITPK1. Reactions were either incubated overnight or as specified in the text.

Assays with [^32^P]-Ins3P substrate were performed under regeneration conditions, with 2.5 mM substrate, 1mM ATP, 15 mM phosphocreatine with 1.26 μM protein(s) for 30 min, 1 h, 2 h, 4 h or 6 h. An aliquot (30 μL) of a hydrolysate of InsP_6_ was added immediately prior to HPLC injection with the sample made up to 50 μL with water.

Assays with Ins3P and [^32^P]-ATP substrate were performed under regeneration conditions, with 2.5 mM substrate, 1mM ATP, 1850 kBq [γ-^32^P]-ATP, 15 mM phosphocreatine with 1.26 μM protein(s) for 30 min, 1 h, 4 h, 8 h or 24 h. An aliquot (30 μL) of a hydrolysate of InsP_6_ was added immediately prior to HPLC injection with the sample made up to 50 μL with water.

Reactions were stopped by the addition of an equal volume of 60 mM (NH_4_)_2_HPO_4_, pH 3.35, or, for suppressed ion-conductivity analysis, by the addition of 3 volumes of 20 mM disodium EDTA, 100 mM NaF. Products were centrifuged at 14,000x *g* for 5 minutes and transferred to autosampler vials with approximately 50-70% of the reaction products injected.

#### Inositol phosphates

Compounds were obtained from Cayman Chemicals or from other sources described in (28,29,50). Synthetic inositol phosphates were purified by ion-exchange chromatography, eluting with a gradient of triethylammonium bicarbonate and were fully characterised as their triethylammonium salts by 1H and 31P NMR spectroscopy and MS and were shown by HPLC to be >95% pure.

#### HPLC of inositol phosphates

#### CarboPacPA200

Products of enzyme assays were resolved by anion exchange on CarboPac PA200 (Dionex) eluted with methanesulfonic acid or HCl and were detected by complexation with ferric ion (28). For some assays, the gradient of methanesulfonic acid employed was changed: time (min), % B (0.6 M MeSA); 0,0; 25,25; 100,38; 45,100.

#### AS11

To compare rates of phosphorylation of Ins1P and Ins3P, assay products were also analysed by suppressed-ion conductivity on a Dionex (UK) ICS-2100 system after resolution on a 250x2 mm AS11 (Dionex) column with 50x2 mm AG11 (Dionex) guard column eluted at a flow rate of 0.35 mL min^-1^ with KOH. The gradient: time (min), KOH (mM); 0,5; 40, 80; was delivered with gradient function ‘5’ in the Chromeleon v.6 software (Dionex, UK) with the anion suppressor current set at 99 mA and column oven at 30°C. The column was washed with 5 mM KOH for 10 minutes between injections. Ins1P and Ins3P stocks (Cayman Chemicals) were initially analysed on a gradient: time (min), KOH (mM); 0,5; 80,80; to normalize concentrations before inclusion at 500 μM in enzyme assay. While the injected sample contained components that obscure the substrate peak and that of potential InsP_2_ and InsP_3_ products, viz. buffer components and ‘stopping’ reagents, baseline resolution of phosphate, ATP and InsP_4_ and Ins(1,3,4,5,6)P_5_ products was obtained. The absence of appreciable accumulation of InsP_2_ and InsP_3_ products was verified in the same samples by post-column detection with ferric nitrate for which the ‘stopping’ reagents do not interfere.

The Ins(1,3,4,5,6)P_5_ product peak (of Figure 7) was integrated with Chromeleon software, referenced to Ins(1,3,4,5,6)P_5_ standard. The rate and standard error of the measurement from single determinations at each time point was obtained from the regression line of the time course, taking account of protein content of the assay.

#### Partisphere SAX

Radiometric assays were analysed by on-line Cerenkov counting in either a Radiomatic A515 series (Canberra Packard, UK) or a β-Ram 5 (LabLogic, UK) flow-detector, both fitted with a 500 μL flow cell, using an integration interval of 1 s. For these assays, reaction products were resolved on a 250x4.6 mm Partisphere SAX (Whatman, obtained from Hichrom, UK) column maintained at 30°C and eluted at 0.8 mL min^-1^. The gradient, made from water [A] and 1.25 M (NH_4_)_2_HPO_4_, pH 3.8 adjusted with H_3_PO_4_ [B], was constructed: time (min), % B; 0,0; 5,0; 65,100; 75,100. A UV detector placed upstream of the radio-detector was set at 254 nm to monitor elution of nucleotide; the signal also reports development of the gradient. Peaks were integrated with the Flo-One for Windows software of the flow-detector, with the Ins(1,3,4,5,6)P_5_ peak (of Figure 5) expressed as a % of counts recovered in the summed peaks of the gradient. The standard error of the linear regression as calculated by GraphPad Prism v.6 yielded a rate constant of 19.42±0.800 nmol min^-1^ mg^-1^ (0.70±0.029 min^-1)^, from single determinations at each time point. The calculation takes account of protein content of the assay and starting substrate concentration.

All HPLC data was exported as ASCII files and re-plotted in GraphPad Prism v6.0 without data smoothing or manipulation, other than offset on the *y*-scale of individual figures to aid clarity.

#### Measurement of inositol

Inositol was separated from sugars by 2d-HPLC on CarboPac PA1 (Dionex) and MA1 (Dionex) columns with detection by pulsed amperometry on the gold electrode of a Dionex DX600 HPLC machine (51). For this, inositol was extracted by grinding of seeds in a liquid nitrogen-chilled 1.5 mL Eppendorf tube using a polypropylene micro-pestle with subsequent extraction for 30 minutes on ice in 300 μL 0.6 M HCl, with further grinding. Samples were diluted 10-20x in 18.2 MOhm cm water before injection of 20 μL aliquots. Peaks were integrated in Chromeleon software and quantified against a calibration curve of 10-200 pmol injections in 20 μL.

#### Genotypes

Genotypes employed in this study were described (27) or were obtained by genetic crosses of genotypes described therein.

## Supplementary Material

Supplementary

## Abbreviations

*Af*IPS: *Archeoglobus fulgidus* Inositol Phosphate Synthase
*At*IPK1: *Arabidopsis thaliana* inositol pentakisphosphate 2-kinase
*At*ITPK1: *Arabidopsis thaliana* inositol tris/tetrakisphosphate kinase 1
DTT: reduced dithiothreitol
EDTA: ethylenediamine tetra-acetic acid
HEPES: 4-(2-hydroxyethyl)-1-piperazineethane sulfonic acid
His: histidine
HPLC: high-pressure liquid chromatography
IP3-3K: inositol 1,4,5-trisphosphate 3-kinase
IP6K: inositol hexakisphosphate kinase
IPMK (IPK2): inositol polyphosphate multikinase
Ins1P: 1D-*myo*-inositol 1-monophosphate
Ins3P: 1D-*myo*-inositol 3-monophosphate
Ins4P: 1D-*myo*-inositol 4-monophosphate
Ins(1,3)P_2_: *myo* (meso)-inositol 1,3-bisphosphate
Ins(1,4)P_2_: 1D-*myo*-inositol 1,4-bisphosphate
Ins(1,5)P_2_: 1D-*myo*-inositol 1,5-bisphosphate
Ins(3,4)P_2_: 1D-*myo*-inositol 3,4-bisphosphate
Ins(4,5)P_2_: 1D-*myo*-inositol 4,5-bisphosphate
Ins(1,3,4)P_3_: 1D-*myo*-inositol 1,3,4-trisphosphate
Ins(1,3,5)P_3_: *myo*(meso)-inositol 1,3,5-trisphosphate
Ins(1,4,5)P_3_: 1D-*myo*-inositol 1,4,5-trisphosphate
Ins(1,4,6)P_3_: 1D-*myo*-inositol 1,4,6-trisphosphate
Ins(3,4,5)P_3_: 1D-*myo*-inositol 3,4,5-trisphosphate
Ins(3,4,6)P_3_: 1D-*myo*-inositol 3,4,6-trisphosphate
Ins(1,3,4,5)P_4_: 1D-*myo*-inositol 1,3,4,5-tetrakisphosphate
Ins(1,3,4,6)P_4_: *myo*-inositol 1,3,4,6-tetrakisphosphate
Ins(1,3,5,6)P_4_: 1D*-myo*-inositol 1,3,5,6-tetrakisphosphate
Ins(1,4,5,6)P_4_: 1D-*myo*-inositol 1,4,5,6-tetrakisphosphate
Ins(3,4,5,6)P_4_: 1D-*myo*-inositol 3,4,5,6-tetrakisphosphate
Ins(1,3,4,5,6)P_5_: *myo*-inositol 1,3,4,5,6-pentakisphosphate
InsP_6_, Ins(1,2,3,4,5,6)P_6_: *myo*-inositol 1,2,3,4,5,6-hexakisphosphate
5-InsP_7_: 5-PP-InsP_5_
IPTG: isopropyl β-D-1-thiogalactopyranoside, MIPS, 1D*-myo*-inositol 3-phosphate synthase
Ni-NTA: nickel-nitriloacetic acid
P_i_: orthophosphate
*St*ITPK1: *Solanum tuberosum* inositol tris/tetrakisphosphate kinase
Tris: tris(hydroxymethyl)aminomethane
VIH1: *Arabidopsis thaliana* diphosphoinositol pentakisphosphate kinase 1
VIH2: *Arabidopsis thaliana* diphosphoinositol pentakisphosphate kinase 2
