# A minimum catalytic unit for synthesis of InsP_6_ and 5-PP-InsP_5_ in *Arabidopsis*

**DOI:** 10.1042/BCJ20253161

**Published:** 2025-12-17

**Authors:** Hayley L. Whitfield, Colleen Sprigg, Andrew M. Riley, Barry V.L. Potter, Hui-Fen Kuo, Charles A. Brearley

**Affiliations:** 1 School of Biological Sciences, University of East Anglia, Norwich Research Park, Norwich, NR4 7TJ, U.K.; 2 Medicinal Chemistry & Drug Discovery, Department of Pharmacology, University of Oxford, Oxford, OX1 3QT, U.K.; 3 Agricultural Biotechnology Research Centre, Academia Sinica, Taipei, 115, Taiwan

**Keywords:** cell biology, inositol, phosphate

## Abstract

Inositol pyrophosphates (diphosphoinositol phosphates) are reported agents of phosphate homeostasis, disease resistance, and hormone action in plants. Of the enzymes that have been shown to synthesize inositol pyrophosphates, inositol tris/tetrakisphosphate (ITPK)1 and *Arabidopsis thaliana* diphosphoinositol pentakisphosphate kinase (VIH)1/VIH2 share the ATP-grasp fold—the latter also possesses a phosphatase domain. Among ATP-grasp inositol phosphate kinases, ITPK1 is particularly flexible—phosphorylating equatorial hydroxyls and equatorial phosphates on inositol phosphates. Herein, we show that the combination of ITPK1 and inositol pentakisphosphate 2-kinase (IPK1) is sufficient to synthesize 5-PP-InsP_5_ from 1D-*myo*-inositol 3-monophosphate (Ins3P) and that ITPK1 is capable of converting 1D-*myo*-inositol 1-monophosphate to myo-inositol 1,3,4,5,6-pentakisphosphate [Ins(1,3,4,5,6)P_5_]. In defining a minimal catalytic unit for synthesis of both *myo*-inositol 1,2,3,4,5,6-hexakisphosphate (InsP_6_/Ins(1,2,3,4,5,6)P_6_) and 5-PP-InsP_5_, we define the minimum enzymology of the ‘lipid-independent’ pathway of InsP_6_ synthesis from Ins3P and its intermediates. The pathway proceeds: Ins3P, 1D-*myo*-inositol 3,4-bisphosphate, 1D-*myo*-inositol 3,4,5-trisphosphate, 1D-*myo*-inositol 3,4,5,6-tetrakisphosphate, Ins(1,3,4,5,6)P_5_, Ins(1,2,3,4,5,6)P_6_, and therefrom to 5-diphosphoinositol-1,2,3,4,6-pentakisphosphate [5-PP-Ins(1,2,3,4,6)P_5_].

## Introduction

Plants, animals, and yeast possess orthologs of D-*myo*-inositol 3-phosphate synthase (MIPS, also known as IPS or ISYN) that catalyzes the cyclo-aldolization of D-glucose 6-phosphate to D-Ins3P [1]. As such, inositol phosphate metabolism is one step removed from glycolysis. Dephosphorylation of 1D-*myo*-inositol 3-phosphate (Ins3P) provides inositol that is incorporated into phosphatidylinositol, and therefrom to phosphatidylinositol phosphates, by phosphatidylinositol synthase. In *Saccharomyces cerevisiae*, phospholipase C, inositol polyphosphate multikinase (IPK2/IPMK, ARG82), and inositol pentakisphosphate 2-kinase (IPK1) comprise a lipid-dependent pathway that synthesizes *myo*-inositol 1,2,3,4,5,6-hexakisphosphate (InsP_6_) from the phosphoinositide Ptd-1D-*myo*-inositol 4,5-bisphosphate [PtdIns(4,5)P_2_] via 1D-*myo*-inositol 1,4,5-trisphosphate [Ins(1,4,5)P_3_], 1D-*myo*-inositol 1,3,4,5-tetrakisphosphate [Ins(1,3,4,5)P_4_]/1D-*myo*-inositol 1,4,5,6-tetrakisphosphate [Ins(1,4,5,6)P_4_], and *myo*-inositol 1,3,4,5,6-pentakisphosphate [Ins(1,3,4,5,6)P_5_] intermediates [2]. The same pathway was also described in *Schizosaccharomyces pombe* [3]. By contrast, a lipid-independent pathway of InsP_6_ synthesis starting with inositol was described in the protist *Dictyostelium discoideum* [4] and duckweed [5–7]. The extent to which the two canonical pathways intersect is poorly defined. Nevertheless, a single point of consensus across varied taxa is that phosphorylation of the axial 2-OH of Ins(1,3,4,5,6)P_5_ is the predominant final step of InsP_6_ synthesis in *Dictyostelium*, fungi, plants, and animals, whether by nuclear or cytosolic activities [2,4,8, reviewed 9]. It is unknown, however, whether Ins(1,3,4,5,6)P_5_ is the only intermediate shared between lipid-dependent and lipid-independent pathways [10].

Yeast lacks the inositol tris/tetrakisphosphate (ITPK) family of ATP-grasp kinases present in plants and animals, while both yeast and plants lack inositol 1,4,5-trisphosphate 3-kinase that phosphorylates the 3-hydroxyl of Ins(1,4,5)P_3_, which is generated following cell-surface receptor activation in animals [9]. Though not widely appreciated, a route of Ins(1,3,4,5,6)P_5_ synthesis from 1D-*myo*-inositol 3,4,6-trisphosphate [Ins(3,4,6)P_3_] was described in avian erythrocytes [11], in which InsP_6_ is a minor component [12]. Moreover, angiotensin-stimulated generation of 1D-*myo*-inositol 3,4,5,6-tetrakisphosphate [Ins(3,4,5,6)P_4_] and subsequent conversion to Ins(1,3,4,5,6)P_5_ [13,14] made it likely that enzymes other than PLC, IPK2, and IPK1 participate in InsP_6_ synthesis in animals (reviewed 10). Similar conclusions were reached in early genetic studies of inositol phosphate synthesis in plants [15,16]. This proposition was, however, only recently demonstrated formally in animals by ^3^H-inositol labeling of ITPK1 knock-outs in human HT-29 and HCT116 cells, albeit without identification of isomers [17,18]. Notwithstanding all the above, the intermediates by which inositol monophosphates are converted by known enzymes to InsP_6_ remain an unanswered question, one that offers potential molecular explanation of diverse phenomena.

In plants, the ITPK family has diversified in structure, catalytic activity, and physiological function [19–22]. Disruption of family members reduces InsP_6_ accumulation in species from taxa that include maize [15], rice [23,24], oilseed rape [20,25], and *Arabidopsis* [26,27]. Because substitution of the inositol ring underpins the specificity of inositol phosphate function, we have started by investigating the catalytic repertoire of the *Arabidopsis thaliana* ITPK1 (*At*ITPK1), and inquiring whether disruption of ITPK1 has broader effects on inositol metabolism. Herein, we describe intermediates of phosphorylation of Ins3P to Ins(1,3,4,5,6)P_5_ by *At*ITPK1, production of InsP_6_ therefrom by *A. thaliana* IPK1 (*At*IPK1), and further phosphorylation to 5-PP-InsP_5_ by *At*ITPK1. We show, as in other species [23,24], that disruption of ITPK1 increases inositol levels in *Arabidopsis*.

## Results

### Successive phosphorylation of Ins3P by *At*ITPK1

**Figure 1:**
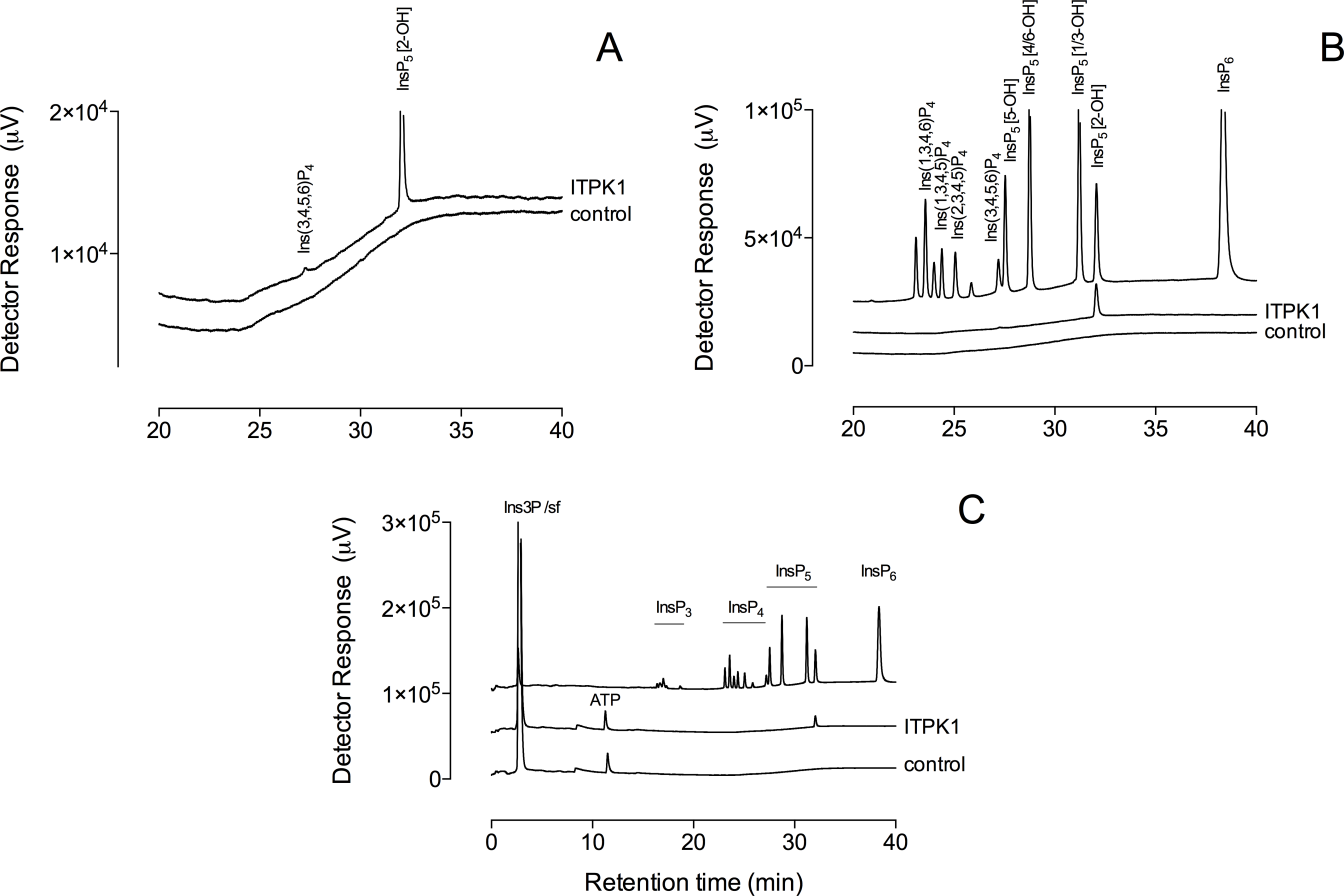
ITPK1 converts Ins3P to Ins(1,3,4,5,6)P_5_. Products of the reaction of Ins3P with ITPK1 and ATP were resolved by HPLC on CarboPac PA200 eluted with methanesulfonic acid. Inositol phosphates were detected with ferric ion. (**A**) An expanded view of the elution of InsP_4_ and InsP_5_ peaks in reaction products. (**B**) An expanded view of the elution of InsP_4_ and InsP_5_ isomers, some of especial relevance to the ensuing discussion: Ins(1,3,4,6)P_4_, Ins(1,3,4,5)P_4_ and Ins(1,3,4,5,6)P_5_ (InsP_5_ [2-OH]), is shown beside products of phosphorylation of Ins3P. (**C**) The position of elution of broad classes of inositol phosphate, viz. InsP_3_, InsP_4_, InsP_5_ and InsP_6_, and ATP is shown. Ins3P elutes with the solvent front (sf). This chromatography has been repeated on more than 30 occasions, with Ins(1,3,4,5,6)P_5_ the single InsP_5_ product. The single *x*-axis label of panel C applies to panels A and B. Ins(1,3,4,6)P_4_, *myo*-inositol 1,3,4,6-tetrakisphosphate; sf, solvent front.

Previous characterization of *At*ITPK1 and *Solanum tuberosum* ITPK1 (*St*ITPK1) [28,29] shows that plant ITPK1 has robust Ins(3,4,5,6)P_4_ 1-hydroxykinase activity, 2 to 3 orders of magnitude greater than its InsP_6_ or PP-InsP_5_ phosphokinase activity [29–32]. Here, *At*ITPK1 converted Ins3P to the *meso*-compound Ins(1,3,4,5,6)P_5_, along with a minor peak of intermediate level of phosphorylation that co-eluted with Ins(3,4,5,6)P_4_ (Figure 1). Even though Ins(1,4,5,6)P_4_ is not separable from its enantiomer Ins(3,4,5,6)P_4_ on non-chiral chromatography, the simplest explanation of InsP_4_ production is that the 3-phosphate is retained. The alternative possibility that the 3-phosphate is removed and replaced with a phosphate in the 1-position, while more involved, would allow for conversion to Ins(1,4,5,6)P_4_ that co-elutes with Ins(3,4,5,6)P_4_.

**Figure 2:**
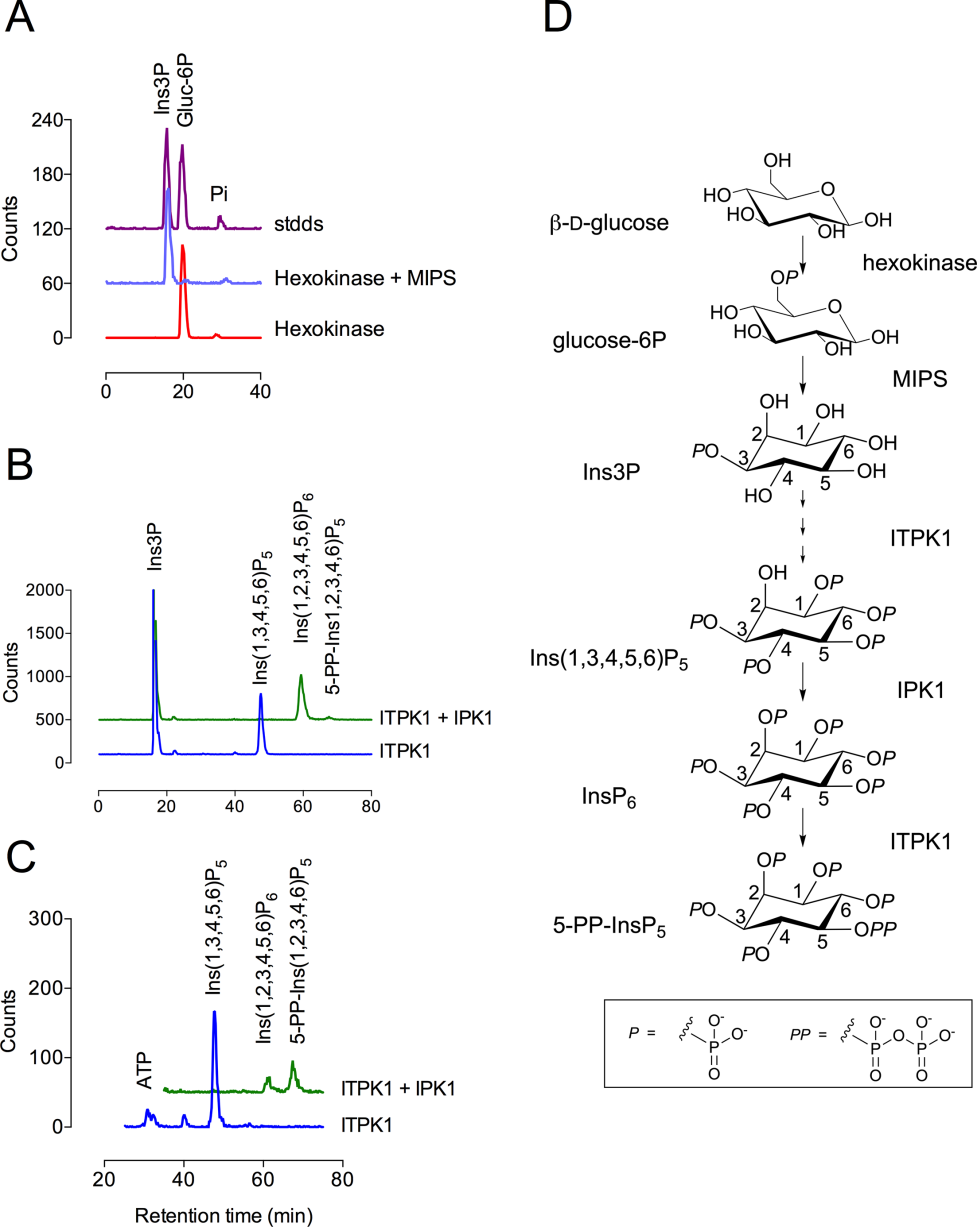
A four-enzyme conversion of glucose to 5-PP-InsP_5_. (**A**) HPLC of conversion of D-glucose to [^32^P]-D-glucose 6-phosphate with hexokinase and [γ-^32^P]-ATP (red trace), and therefrom to [^32^P]-Ins3P with MIPS (blue trace), with HPLC of a mixed sample (purple trace). Products resolved on Partisphere SAX eluted with 40 mM [NH_4_]_2_HPO_4_. (**B**) HPLC of conversion of [^32^P]-Ins3P to Ins(1,[^32^P]3,4,5,6)P_5_ with ITPK1 (blue trace) and, by inclusion of IPK1, to 5-PP-Ins(1,2,[^32^P]3,4,6)P_5_ (green trace). Products resolved on Partisphere SAX eluted with a gradient of [NH_4_]_2_HPO_4_. (**C**) HPLC of conversion of unlabeled Ins(1,4,5,6)P_4_ to Ins(1,[^32^P]3,4,5,6)P_5_ with ITPK1 and [γ-^32^P]-ATP (blue trace), and therefrom to [^32^P]-InsP_6_ and [^32^P]-5-PP-Ins(1,2,3,4,6)P_5_ by addition of IPK1 (green trace). HPLC on Partisphere SAX eluted as B. In B and C, the ^32^P label added into the 3-position of Ins(1,3,4,5,6)P_5_ (from [γ-^32^P]-ATP) is retained in InsP_6_ and 5-PP-InsP_5_ products. (**D**) Summary of synthesis scheme. The single *x*-axis label of panel C applies to panels A and B.

To prove retention of the 3-phosphate and remove any ambiguity surrounding the regiomeric character of 1- vs 3-phosphates in intermediates, [^32^P]-Ins3P was synthesized. [^32^P]-glucose 6-phosphate was synthesized from glucose and [γ-^32^P]-ATP with yeast hexokinase. Subsequently, by driving the reaction with excess glucose and an ATP-regenerating system, more than 95% of the ATP was converted (Figure 2A). Using thermostable MIPS from *Archaeoglobus fulgidus*, [^32^P]-glucose 6-phosphate was converted to [^32^P]-Ins3P (Figure 2A).

**Figure 3:**
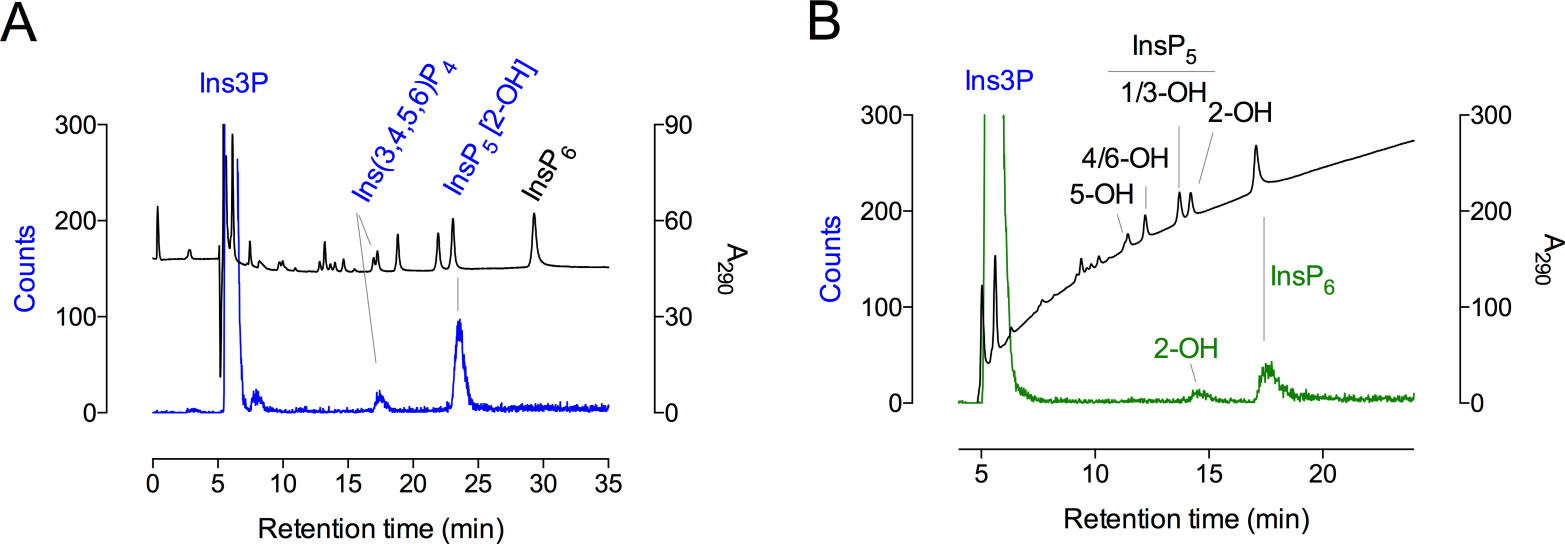
Retention of ^32^P label during conversion of Ins3P to InsP_6_ via Ins(3,4,5,6)P_4_ and Ins(1,3,4,5,6)P_5_. (**A**) HPLC on CarboPac PA200 eluted with methanesulfonic acid of products from the assay of [^32^P]-Ins3P with ITPK1 (blue trace). (**B**) Products from the assay with ITPK1 and IPK1 (green trace) were eluted with HCl. Elution of inositol phosphate standards spiked into the samples and detected with ferric ion are shown (black traces).

Addition of ITPK1 and an ATP-regenerating system allowed phosphorylation of [^32^P]-Ins3P to ^32^P-labeled InsP_4_ and InsP_5_ products, resolved on different HPLC phases (**Figures 2B, 3A and B**). The precise elution of [^32^P]-InsP_4_ with Ins(1,4,5,6)P_4_/Ins(3,4,5,6)P_4_ and retention of the ^32^P-labeling unambiguously identifies Ins(3,4,5,6)P_4_ as an intermediate in the conversion of Ins3P to Ins(1,3,4,5,6)P_5_. Previously, the products of phosphorylation of Ins(3,4,5,6)P_4_ by the ITPK1/IPK1 couple were shown to be Ins(1,3,4,5,6)P_5_ (InsP_5_ [2-OH]), InsP_6_, and 5-PP-InsP_5_ (5-InsP_7_) [28]. Thus, from Ins(3,4,5,6)P_4_ all successive phosphorylations generate achiral (*meso*-) products. In the current study, inclusion of IPK1 allowed retention of the ^32^P-labeled 3-phosphate (of Ins3P) in InsP_6_ (**Figures 2B, D, 3B**) and 5-PP-InsP_5_ (Figure 2B and D). Ins(1,4,5,6)P_4_ is a weaker substrate of ITPK1 than its enantiomer Ins(3,4,5,6)P_4_ [26,27], and its use here confirmed that addition of a 3-phosphate is retained in the InsP_6_ and 5-PP-InsP_5_ products of the combined action of ITPK1 and IPK1 (Figure 2C).

### Identification of intermediates in the conversion of Ins3P to Ins(1,3,4,5,6)P_5_

Except for the generation of a peak with the mobility of Ins(3,4,5,6)P_4_, radiolabeled peaks representing intermediates in phosphorylation between InsP and InsP_4_ were trivial components and not reliably observed. Similar conclusions can be drawn for human ITPK1 [18]. Chromatography on CarboPac PA200 similarly failed to reveal peaks with chromatographic properties of InsP_2_ and InsP_3_ (Figure 1).

Taking a candidate product/intermediate approach to phosphorylation of Ins3P, phosphorylation of the axial 2-hydroxyl can be discounted for two reasons: ITPK1 does not add a 2-phosphate to a wide range of tested substrates [28,29], and also because addition of IPK1 is required to phosphorylate the ‘vacant’ 2-hydroxyl of the [^32^P]-InsP_5_ product later in the sequence (**Figures 2C and 3B**). The only enzyme yet shown to possess this activity across plant, metazoan, and fungal taxa is IPK1, which has a different structural fold. Similarly, *myo*(*meso*)-inositol 1,3-bisphosphate [Ins(1,3)P_2_] can be discounted as a product because the 1-phosphate is added later, in the conversion of Ins(3,4,5,6)P_4_ to Ins(1,3,4,5,6)P_5_ (**Figures 2B, D, 3A**). This leaves the three ‘undetected’ possible InsP_2_ products of phosphorylation of Ins3P as 1D-*myo*-inositol 3,4-bisphosphate [Ins(3,4)P_2_], Ins(3,5)P_2_, and/or Ins(3,6)P_2_. By similar principles, the InsP_3_ product could be 1D-*myo*-inositol 3,4,5-trisphosphate [Ins(3,4,5)P_3_] or Ins(3,4,6)P_3_, but not Ins(4,5,6)P_3_.

**Figure 4:**
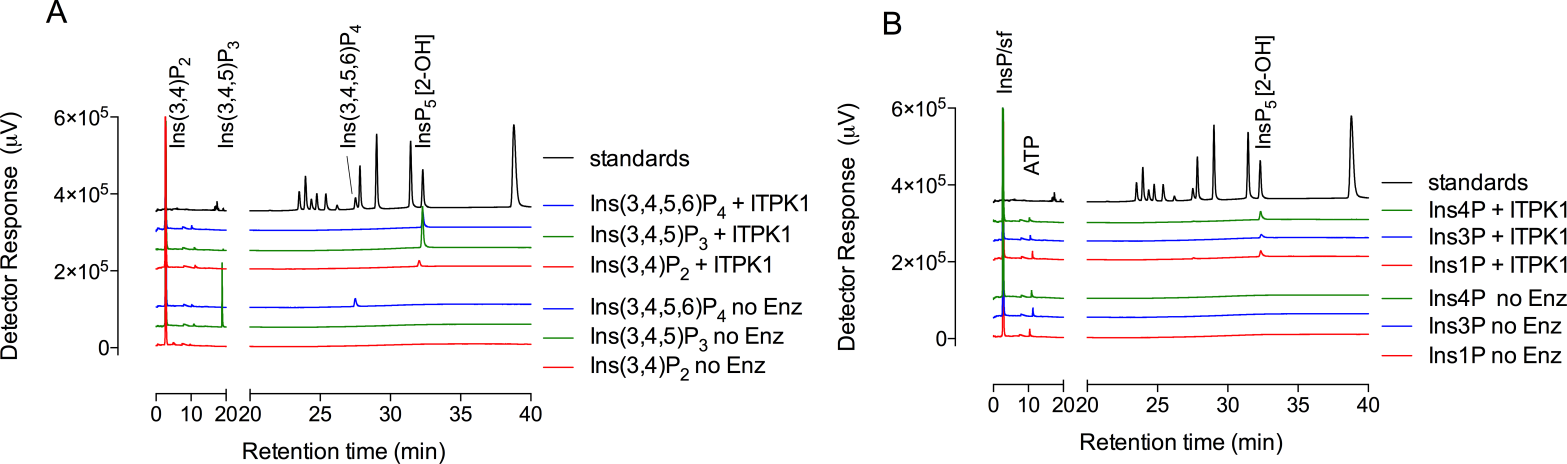
ITPK1 converts inositol monophosphates to Ins(1,3,4,5,6)P_5_. (**A**) Products of a 60-min assay of ITPK1, 200 μM ATP, and 100 μM inositol phosphate were resolved by CarboPac PA200 HPLC eluted with methanesulfonic acid, with detection with ferric ion: Ins(3,4)P_2_ (red trace); Ins(3,4,5)P_3_ (green trace); Ins(3,4,5,6)P_4_ (blue trace). Control assays incubated for 60 min without enzyme are shown: Ins(3,4)P_2_ (red trace), Ins(3,4,5)P_3_ (green trace), and Ins(3,4,5,6)P_4_ (blue trace). (**B)** Products of a 60-min assay of ITPK, 500 μM ATP, and 500 μM inositol phosphate: Ins1P (red trace); Ins3P (blue trace); Ins4P (green trace). Control assays incubated for 60 min without enzyme are shown: Ins1P (red trace), Ins3P (blue trace), and Ins4P (green trace). Inositol monophosphate substrates elute in the solvent front, sf. In A and B, elution of inositol phosphate standards is shown (black trace). Ins4P, 1D-*myo*-inositol 4-monophosphate; sf, solvent front.

Both Ins(3,4)P_2_ and Ins(3,4,5)P_3_ were converted to Ins(1,3,4,5,6)P_5_ (Figure 4A), while Ins(3,4,6)P_3_ was converted to peaks with the chromatographic properties of *myo*-inositol 1,3,4,6-tetrakisphosphate [Ins(1,3,4,6)P_4_] and Ins(1,3,4,5)P_4_ (Table 1). The absence of commercially available Ins(3,5)P_2_ and Ins(3,6)P_2_ precluded their analysis, but the efficient conversion of Ins(3,4,5)P_3_ is consistent with Ins(3,4)P_2_ as a precursor.

**Table 1:** Products of ITPK1 activity

Substrate	Most phosphorylated product(s)	Reference
Ins1P	Ins(1,3,4,5,6)P_5_	This study
Ins3P	Ins(1,3,4,5,6)P_5_	This study
Ins4P	Ins(1,3,4,5,6)P_5_	This study
Ins(1,3)P_2_	Ins(1,3,4,5,6)P_5_	This study
Ins(1,4)P_2_	Ins(1,3,4,6)P_4_ & Ins(1,3,4,5)P_4_	This study & *
Ins(1,5)P_2_	Ins(1,3,4,5,6)P_5_	This study
Ins(3,4)P_2_	Ins(1,3,4,5,6)P_5_	This study
Ins(4,5)P_2_	Ins(1,3,4,5,6)P_5_	This study
Ins(1,3,4)P_3_	Ins(1,3,4,6)P_4_ & Ins(1,3,4,5)P_4_	This study & *
Ins(1,3,5)P_3_	Ins(1,3,4,6)P_4_ & Ins(1,3,4,5)P_4_	This study
Ins(1,4,5)P_3_	Ins(1,3,4,6)P_4_ & Ins(1,3,4,5)P_4_	This study & *
Ins(1,4,6)P_3_	Ins(1,3,4,6)P_4_	This study & *
Ins(3,4,5)P_3_	Ins(1,3,4,5,6)P_5_	This study
Ins(3,4,6)P_3_	Ins(1,3,4,6)P_4_ & Ins(1,3,4,5)P_4_	This study
Ins(1,3,4,5)P_4_	Ins(1,3,4,6)P_4_ & Ins(1,3,4,5)P_4_	This study
Ins(1,3,5,6)P_4_	Ins(1,3,4,5,6)P_5_	This study
Ins(1,4,5,6)P_4_	Ins(1,3,4,5,6)P_5_	This study & *
Ins(3,4,5,6)P_4_	Ins(1,3,4,5,6)P_5_	This study & *
InsP_6_	5-PP-InsP_5_	*
1-PP-InsP_5_	1,5-(PP)_2_-InsP_4_	*
3-PP-InsP_5_	3,5-(PP)_2_-InsP_4_	*

The reactions reported here have been observed at least three times, commonly on tens of occasions. Among products: Ins(1,3,4,6)P_4_ and Ins(1,3,4,5,6)P_5_ are *meso*-compounds, whereas Ins(1,3,4,5)P_4_ is chiral and is not resolvable from the enantiomer Ins(1,3,5,6)P_4_. ITPK1 shows isomerase activity and is able to interconvert Ins(1,3,4,6)P_4_ and Ins(1,3,4,5)P_4_ [33–35].* Reactions were also reported [28,29,31,32].

In summary, *At*ITPK1 is shown to convert Ins3P to Ins(1,3,4,5,6)P_5_ via Ins(3,4)P_2_, Ins(3,4,5)P_3_, and Ins(3,4,5,6)P_4_ intermediates (Figure 4A and B).

### Kinetics of Ins3P phosphorylation

**Figure 5:**
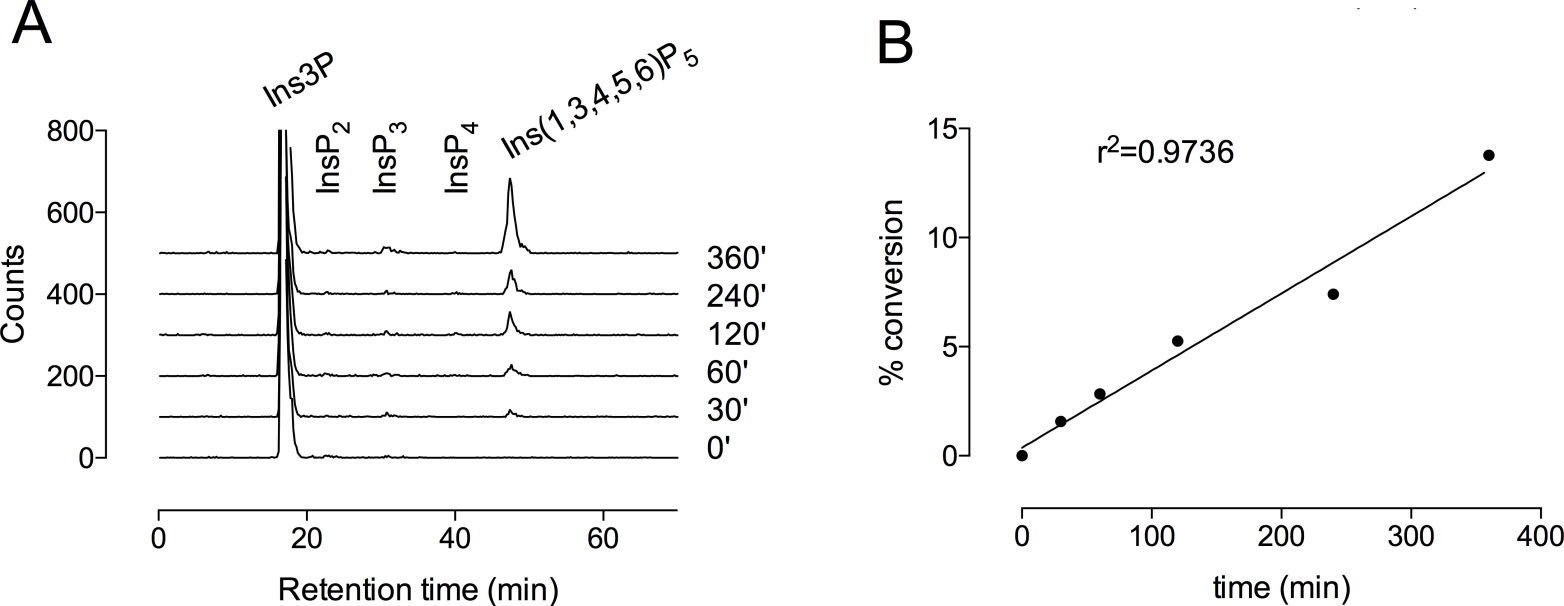
Kinetics of [^32^P]-Ins3P phosphorylation to Ins(1,3,4,5,6)P_5_ by ITPK1. (**A**) Products of reactions were analyzed by HPLC on Partisphere SAX. (**B**) Percentage conversion to Ins(1,3,4,5,6)P_5_.

To determine the kinetics of phosphorylation of Ins3P, we used [^32^P]-Ins3P as a substrate. The appearance of ^32^P-labeled products was followed over a time course. The approach allows us to conclude that the reaction was not substrate limited, with less than 14% conversion of starting material at the final time point (Figure 5A and B). The standard error of the linear regression as calculated by GraphPad Prism v.6 yielded a rate constant of 19.42 ± 0.800 nmol min^-1^ mg^-1^ (0.70 ± 0.029 min^-1^). This value is similar to that with InsP_6_ substrate but is a small fraction of that obtained with Ins(3,4,5,6)P_4_ [28]. At 6 h of reaction, a small peak of a putative [^32^P]-InsP_3_ was evident on Partisphere SAX (Figure 5A).

### Phosphorylation of Ins1P

*In vitro*, human ITPK1 phosphorylates 1D-*myo*-inositol 1-monophosphate (Ins1P), as it does Ins3P, via unknown intermediates to unidentified InsP_5_ product(s) [18]. *At*ITPK1 was tested for phosphorylation of Ins1P, the enantiomer of Ins3P. Ins1P was converted to Ins(1,3,4,5,6)P_5_ (Table 1, Figure 4B). Similarly, Ins(1,3)P_2_, Ins (1,5)P_2_, and 1D-*myo*-inositol 1,3,5,6-tetrakisphosphate [Ins(1,3,5,6)P_4_] were also converted to Ins(1,3,4,5,6)P_5_ (Table 1). Close inspection of our previous study (Figure 3D of [28]) revealed the production of a very small peak of Ins(1,3,4,5,6)P_5_ from Ins(1,3,5,6)P_4_, albeit trivial compared with that generated from Ins(3,4,5,6)P_4_. Here, various isomers of InsP_2_ and InsP_3_ were also tested as potential intermediates. Among these, 1D-*myo*-inositol 1,4-bisphosphate [Ins(1,4)P_2_], 1D-*myo*-inositol 1,3,4-trisphosphate [Ins(1,3,4)P_3_], *myo*(*meso*)-inositol 1,3,5-trisphosphate [Ins(1,3,5)P_3_], Ins(1,4,5)P_3_, and Ins(3,4,6)P_3_ were converted to Ins(1,3,4,6)P_4_ and/or Ins(1,3,4,5)P_4_ but not to Ins(1,4,5,6)P_4_, Ins(3,4,5,6)P_4_, or Ins(1,3,4,5,6)P_5_ (Table 1). Ins(1,3,4,5)P_4_ was interconverted (isomerized [33–35]) to Ins(1,3,4,6)P_4_ without further phosphorylation (Table 1). In a previous study with assays at higher substrate concentration, we did not observe product from Ins(1,3,4,5)P_4_ or from Ins(1,3,4,6)P_4_ [28].

**Figure 6:**
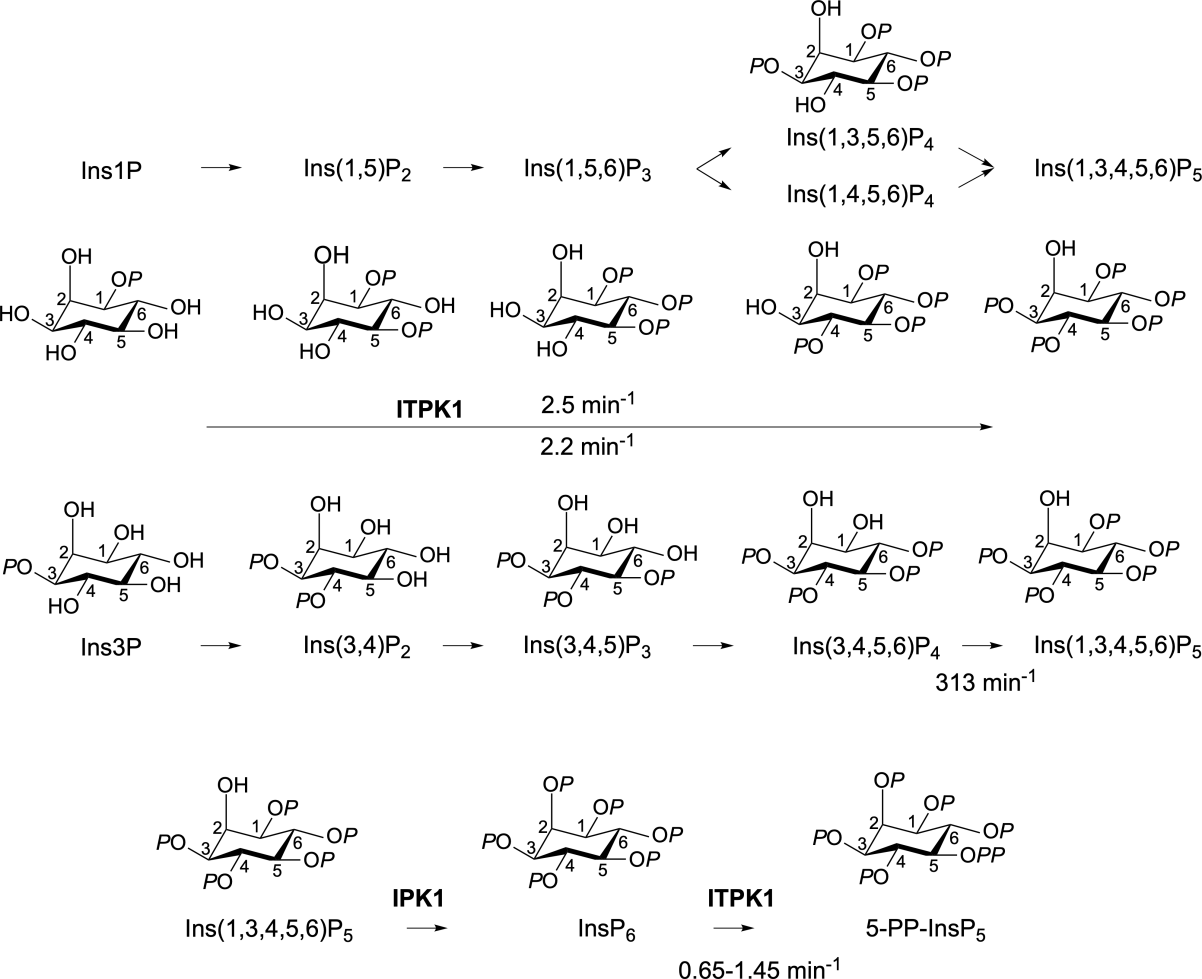
The contribution of ITPK1 to inositol phosphate synthesis. Rate constants for individual and aggregate steps are indicated. For Ins1P, the involvement of Ins(1,3,6)P_3_ and Ins(1,5,6)P_3_ are speculations based on demonstrated conversion of Ins(1,3)P_2_ and Ins(1,5)P_2_, and of Ins(1,3,5,6)P_4_ and Ins(1,4,5,6)P_4_, to Ins(1,3,4,5,6)P_4_ (Table 1 and text). Ins(1,5)P_2_, 1D-*myo*-inositol 1,5-bisphosphate.

Collectively, these data suggest that all potential flux is channeled to Ins(1,3,4,5,6)P_5_ via Ins(1,3,5,6)P_4_ and/or Ins(1,4,5,6)P_4_ for Ins1P and via Ins(3,4,5,6)P_4_ for Ins3P (Figure 6). Without Ins(1,6)P_2_ or Ins(1,3,6)P_3_, we were unable to test these as potential intermediates for phosphorylation of Ins1P, but we speculate that both Ins(1,3,6)P_3_ and Ins(1,5,6)P_3_ are preferred intermediates in the conversion of Ins1P to Ins(1,3,4,5,6)P_5_.

ITPKs have been most intensively studied in relation to InsP_4_ and InsP_5_ turnover [13,14,36–38]. To address how fluxes from lower-order precursors such as inositol monophosphates [10,18] might feed into Ins(1,3,4,5,6)P_5_ synthesis, we also considered 1D-*myo*-inositol 4-monophosphate (Ins4P), even though this monophosphate is considered to be a product of Ins(1,4,5)P_3_ turnover in animal cells [9]. Indeed, Ins4P was also converted to Ins(1,3,4,5,6)P_5_ (Table 1, Figure 4B). Given the inability of ITPK1 to convert Ins(1,4)P_2_ to Ins(1,4,5,6)P_4_ or Ins(1,3,4,5,6)P_5_, the absence of 2-hydroxykinase activity, the phosphorylation of Ins(3,4)P_2_ to Ins(1,3,4,5,6)P_5_, the conversion of Ins(4,5)P_2_ to Ins(1,3,4,5,6)P_5_ and of Ins(3,4,6)P_3_ only as far as Ins(1,3,4,6)P_4_ and Ins(1,3,4,5)P_4_ (Table 1), we speculate that Ins4P phosphorylation proceeds via Ins(3,4)P_2_ and/or Ins(4,5)P_2_, Ins(3,4,5)P_3_, and/or Ins(3,4,5,6)P_4_ to Ins(1,3,4,5,6)P_5_. Without Ins(4,6)P_2_, we were unable to test its potential contribution to Ins(1,3,4,5,6)P_5_ synthesis, but we note that neither 1D-*myo*-inositol 1,4,6-trisphosphate [Ins(1,4,6)P_3_] nor Ins(3,4,6)P_3_ yielded InsP_5_ product (Table 1).

In considering experiments such as these, we comment that where more than one product is generated at a particular level of intermediary phosphorylation, the most prominent intermediate is most likely the weaker substrate (i.e. the one left behind in the conversion of the better substrate to a ‘higher’ product). Indeed, in the scenario of ITPK1 action against Ins(1,4,5,6)P_4_ and Ins(3,4,5,6)P_4_, the former is the weakest substrate [28,29]. It is possible that Ins(1,3,5,6)P_4_ is a better substrate than Ins(1,4,5,6)P_4_, and so when monophosphates are converted ‘all the way’ to Ins(1,3,4,5,6)P_5_, the only InsP_4_ peaks observed have the mobility of the Ins(1,4,5,6)P_4_/Ins(3,4,5,6)P_4_ enantiomeric pair.

### Comparison of Ins1P and Ins3P phosphorylation

**Figure 7:**
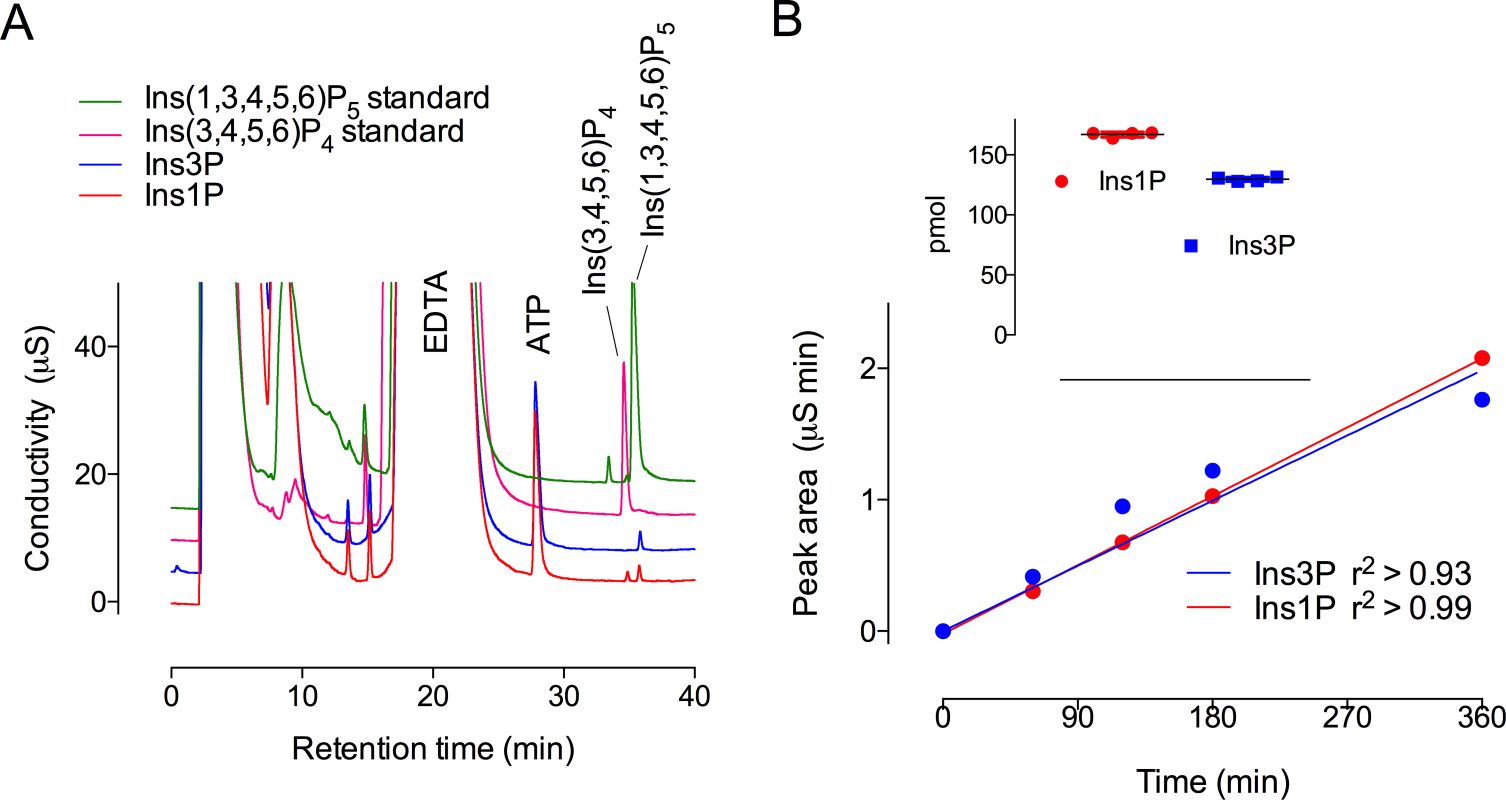
Suppressed ion-conductivity analysis of phosphorylation of Ins1P and Ins3P by ITPK1. Assays stopped by the addition of NaF/EDTA, pH 10 were eluted on a Dionex AS11 column. (**A)** Products generated from Ins1P (red trace) and Ins3P (blue trace) share retention time with Ins(3,4,5,6)P_4_ (cerise trace) and/or Ins(1,3,4,5,6)P_5_ (green trace) standards. The positions of elution of ATP and EDTA are shown. The chromatography shown in the figure, separation of Ins3P (or enantiomer Ins1P) from Ins(3,4,5,6)P_4_ (or enantiomer Ins(1,4,5,6)P_4_) and Ins(1,3,4,5,6)P_5_ has been repeated > 10 times. (**B)** Linearity of reaction with Ins1P and Ins3P from a time-course experiment with single determinations. Inset: four replicate measurements of product formation (InsP_4_ & InsP_5_, summed) from an independent experiment with samples taken at 2 h of incubation.

Direct comparison of the extent of reaction against Ins1P and Ins3P was determined from the production of InsP_4_ and InsP_5_, quantified by conductivity after chromatography on an AS11 column (Figure 7). Phosphorylation of Ins1P generated peaks of InsP_4_, co-eluting with the Ins(1,4,5,6)P_4_/Ins(3,4,5,6)P_4_ enantiomeric pair, and of Ins(1,3,4,5,6)P_5_ (Figure 7A). The same was observed with ferric-based detection on a CarboPac PA200 column (Supplementary Figure S1), whereby the absence of accumulation of InsP_2_ or InsP_3_ intermediates was again confirmed. In contrast, phosphorylation of Ins3P generated a predominant peak of Ins(1,3,4,5,6)P_5_ (Figure 7A and Supplementary Figure S1). The macro constants (for inositol monophosphate conversion to product(s) InsP_4_ and/or InsP_5_) were similar for Ins1P and Ins3P, assayed under conditions in which product accumulation was less than 5% of the starting substrate (Figure 7B). The constants are similar to those reported for InsP_6_ but are 2–3 orders of magnitude smaller than the rate constant for Ins(3,4,5,6)P_4_ phosphorylation (Table 2). These values explain the limited accumulation of InsP_2_ and InsP_3_ intermediates. We posit that ITPK1 ‘gathers pace’ with each successive phosphorylation, possibly in a processive manner, as far as Ins(1,3,4,5,6)P_5_

**Table 2:** Rate constants for substrate phosphorylation by ITPK1*

Substrate	Rate constant (min^-1^) *	Reference
Ins1P	1.46 ± 0.019**	This study
Ins3P	1.14 ± 0.015** (0.70 ± 0.029, ^32^P assay)	This study
Ins(3,4,5,6)P_4_***	313 [8640 nmol min^-1^ mg^-1^]	(28,29)
InsP_6_	1.45 [40 nmol min^-1^ mg^-1^]	(28)
InsP_6_	0.65 [17.87 nmol min^-1^ mg^-1^]	(32)
ADP (from 5-InsP_7_)	1.16 [32.05 nmol min^-1^ mg^-1^]	(32)

* calculated with mass (Da) 36220 (UniProt Q9SBA5)

** macro constant for conversion to InsP_4_ and InsP_5_ products (with value from radiometric assay for Ins3P)

*** for the enantiomer Ins(1,4,5,6)P_4_, the rate constant is a small fraction of that for Ins(3,4,5,6)P_4_ [28,29].

### The metabolic effects of disruption of *Itpk1* extend to inositol

**Figure 8:**
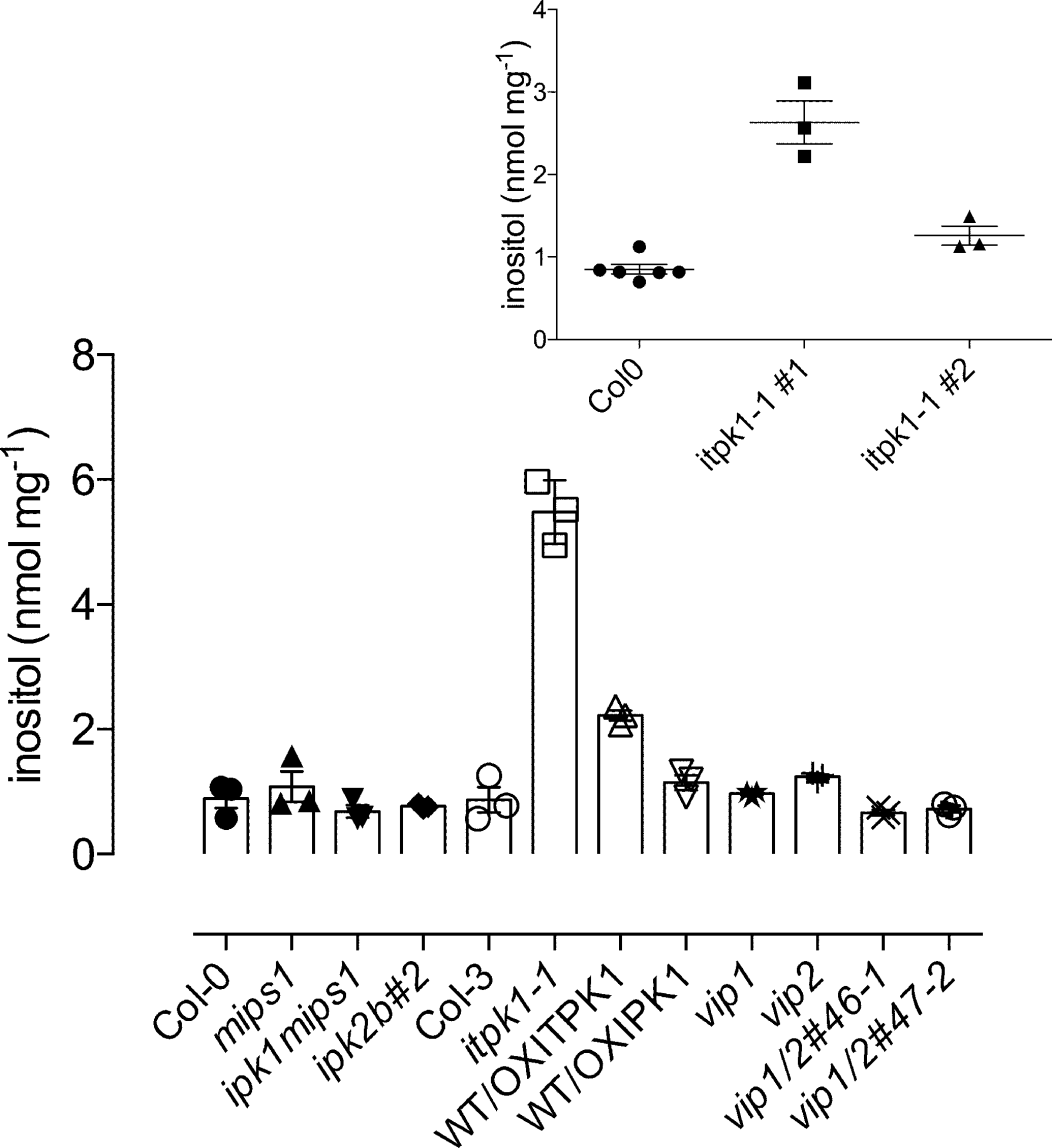
Inositol levels in seeds of various *Arabidopsis* genotypes. Inositol was measured by 2D-HPLC with pulsed amperometric detection, mean and SE of *n* = 3. The genotypes studied include Col0 (WT); *mips1*; a genetic cross between *ipk1* and *mips1*, *ipk1mips1*; *ipk2ß*; Col-3 (WT); *itpk1*; an overexpression line of ITPK1 in WT background, WT/OXITPK1; an overexpression line of IPK1 in WT background, WT/OXIPK1; and different alleles of *vip* mutants. A separate comparison of two *itpk1* lines (*n* = 3) and Col-0 (WT, *n* = 6) is shown in the inset. By two-tailed Student’s T-test, the means were significantly different at *P*<0.05 between Col0 and the *itpk1* lines, both in the main panel and inset.

While varied studies have reported changes in the profile of inositol phosphates in *Arabidopsis* mutants, including *itpk1*, compared with wildtype (WT), and the effect on phosphate homeostasis [27,32,39–42], none has measured inositol. Indeed, while inositol is rarely described in studies of inositol phosphate or inositol pyrophosphate synthesis, it has been observed that disruption of ITPK orthologs not only reduces InsP_6_ but also elevates inositol in rice [23,24]. To test whether inhibition of inositol monophosphate conversion to Ins(1,3,4,5,6)P_5_ elevates inositol in *Arabidopsis*, inositol was measured in seeds of *itpk1* and other mutants of genes involved in inositol polyphosphate and pyrophosphate synthesis (Figure 8). They include *vip* (*A. thaliana* diphosphoinositol pentakisphosphate kinase) mutants widely reported to control phosphate homeostasis (reviewed [39]) and other mutants such as *ipk2ß,* which reduce InsP_6_ levels in seeds and whose WT protein product, combined with IPK1, catalyzes InsP_6_ synthesis from Ins(1,4,5)P_3_ *in vitro* [41]. Disruption of *itpk1* elevated inositol up to 5-fold, while the other genotypes yielded inositol levels similar to WT (Figure 8).

## Discussion

Human ITPK1 synthesizes unidentified InsP_5_ isomer(s) from Ins1P and Ins3P *in vitro,* and deletion of ITPK1 reduces InsP_6_ *in vivo* [17,18]. Expression of human ITPK1 in yeast *plc1*Δ causes the accumulation of InsP_6_, but does so by an unspecified series of intermediates. It was suggested that the InsP_5_ product that is generated in yeast from Ins3P cannot be Ins(1,3,4,5,6)P_5_ on the grounds that expression of human ITPK1 in *plc1*Δ*arg82*Δ yeast did not restore InsP_6_ levels from [^3^H]-inositol [18]. This argument was not validated by independent assignment of identity of the InsP_5_ species and rests also on limited analysis of InsP_3_ and InsP_4_ isomers in WT, mutant, or ‘complemented’ yeast. Indeed, the assignment of identity to inositol phosphates in yeast and in mammalian cells was almost exclusively defined by radiolabeling on Partisphere SAX, which the authors showed [18] to be an incomplete description.

Here, we show that *At*ITPK1 synthesizes Ins(1,3,4,5,6)P_5_ from Ins3P and that inclusion of *At*IPK1 enables accumulation of InsP_6_ and 5-PP-InsP_5_. Thus, ITPK1 and IPK1 constitute a minimum catalytic unit for PP-InsP synthesis in plants. That *At*ITPK1 generates Ins(3,4,5,6)P_4_ from Ins3P is consistent with previous characterizations of *At*ITPK1 and *St*ITPK1 activity towards Ins(3,4,5,6)P_4_ [28,29]. While it is also claimed that mammalian ITPK1 is not responsible for the synthesis of Ins(3,4,5,6)P_4_ from Ins(1,3,4,5,6)P_5_ [18], human ITPK1 has the Ins(3,4,5,6)P_4_ 1-kinase and Ins(1,3,4,5,6)P_5_ 1-phosphatase activities [36,37] that plant ITPK1 possesses [19,34,35]. Metabolic studies also describe Ins(3,4,5,6)P_4_ 1-kinase activities in plants [7,38,43].

It is perhaps remarkable that few have recognized the finely detailed analyses of inositol phosphate metabolism of avian erythrocytes from Stephens and co-workers, particularly [11], that reports Ins(3,4,6)P_3_ to be the precursor of Ins(3,4,5,6)P_4_ and Ins(3,4,5,6)P_4_ to be the principal precursor of Ins(1,3,4,5,6)P_5_. We speculate that these *in vivo* reactions are the manifestation of ITPK1 activity. The precedent also extends to mammals [13,14]. Consequently, we propose that across metazoan taxa, evolution has retained in ITPK1 activity against Ins3P and other lower-order inositol phosphates, but with the highest catalytic activity towards Ins(3,4,5,6)P_4_ [28,29].

The pathway of InsP_6_ synthesis from Ins3P described in Figure 6 for *Arabidopsis* ITPK1 differs only at the InsP_3_ level from that described in *Spirodela polyrhiza* [7]: Ins(3,4,5)P_3_ in the former, Ins(3,4,6)P_3_ in the latter. Few groups have assigned stereoisomerism or enantiomerism to InsP_3_ species in plant tissues: there are 20 possibilities. Consequently, it is difficult to comment further. Also, the intermediates by which ITPK1 contributes to InsP_6_ synthesis in human cell lines are unknown [17,18], excepting the implications of the aforementioned work of Stephens [11], Balla [13,14], and Shears [36,37]. We note, however, that the ITPK class is uniquely diversified in plants [19,20] and that *Spirodela*, a primitive vascular plant with vestigial roots, occupies a clade that appears to have ‘returned to the water’ [44] and which shares an ITPK family structure of four orthologs with *Arabidopsis*.

Compared with WT, *itpk1* seedlings show elevations in ^3^H inositol-labeling of undefined InsP_3_ and InsP_4_ species [27,40] and elevation of labeling of Ins(1,4,5,6)P_4_ and/or Ins(3,4,5,6)P_4_ from ^32^P-orthophosphate [27]. A reduction in ^3^H inositol-labeling of InsP_6_ was observed in the same study, but others [40] describe no such difference between WT and *itpk1*. This disparity possibly has its origins in the different ‘normalizations’ of the two studies. In the latter study, uptake of inositol and labeling of inositol monophosphate are not accounted for, peaks are normalized to counts for peaks eluting thereafter, whereas in the former study, peak areas are normalized to counts across the entire gradient. When comparing genotypes, normalization without considering inositol or all inositol phosphates risks the masking of genotype effect(s) on inositol phosphates and inositol pyrophosphates. Thus, for genotypes such as *mips* [43,45,46] in which phenotype extends to alterations of cellular inositol level (relative to WT) [45], and *itpk1* (this study), changes in specific activity of the inositol pool and subsequent labeling of inositol phosphates are treated differently by the two normalization approaches. Indeed, antisense inhibition of *mips* increased labeling of inositol phosphates from exogenous *myo*-[2-^3^H] inositol four- to five-fold, an observation rationalized by an increase in specific activity of the inositol pool [43].

**Figure 9:**
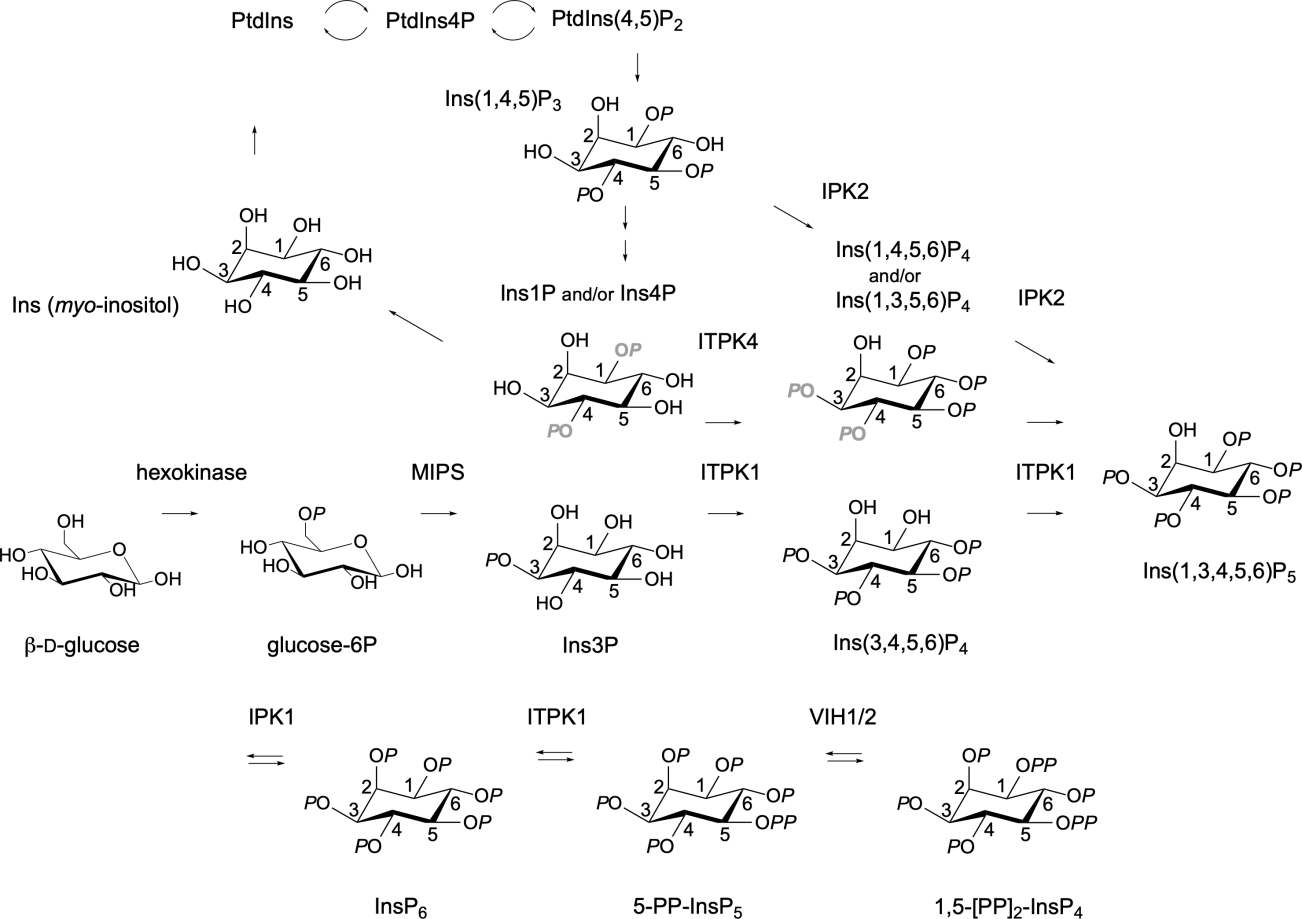
A revised metabolic network for InsP_6_ and PP-InsP synthesis in plants. ITPK1 integrates flux from the inositol monophosphate pool to Ins(1,3,4,5,6)P_5_ and from InsP_6_ to PP-InsPs. Grey letters indicate phosphate positions in the and/or alternatives.

This aside, ITPK1 is not the only ITPK contributor to InsP_6_ synthesis in *Arabidopsis*: *itpk4* mutants show much reduced levels, both of labeling from ^3^H inositol in seedlings [27], of InsP_6_ levels in plants [47] and of seed InsP_6_ levels [26,27]. ITPK4 has reversed specificity for the Ins(1,4,5,6)P_4_/Ins(3,4,5,6)P_4_ enantiomeric pair, favoring Ins(1,4,5,6)P_4_ [21]. Among the family, ITPK1 is also remarkably reversible and preferentially transfers the 1-phosphate of Ins(1,3,4,5,6)P_5_ to ADP, generating Ins(3,4,5,6)P_4_ [21,29]. We posit that ITPK1 is the integrator of flux from Ins3P and potentially also from Ins1P and/or Ins4P to Ins(1,3,4,5,6)P_5_ and therefrom via IPK1 to InsP_6_ whether involving ITPK4 or other inositol phosphate kinases (Figure 9).

In maize [15], rice [23,24], *Arabidopsis* [26,27], and *Brassica napus* [20,25], disruption of ITPK orthologs reduces InsP_6_. This is reminiscent of the effect in seeds and germinating seedlings of disruption of *ipk1* [26,41]. The authors [41] reported that the accumulating InsP_4_ peak comprises predominantly (80%) Ins(3,4,5,6)P_4_. Collectively, these data provide physiological context to the sequential action of ITPK1 and IPK1 in the synthesis of InsP_6_ and of 5-PP-InsP_5_ [28,31], here from Ins3P. They also suggest that only a small part of InsP_6_ accumulation in *Arabidopsis* seeds is independent of ITPK1. The finding that Ins(1,3,5,6)P_4_ is a potential intermediate in the conversion of Ins1P to Ins(1,3,4,5,6)P_5_ is itself an observation that could explain the accumulation of D/L-Ins(1,3,4,5)P_4_ in low-phytate grain mutants, PLP1A, PLP2A, and PLP3A, of barley [48]: Ins(1,3,4,5)P_4_ is the enantiomer of Ins(1,3,5,6)P_4_, the enantiomers are not resolved chromatographically, nor are they distinguished by the NMR characterization [48].

Irrespective of the fine detail of ITPK1 involvement in InsP_6_ synthesis, disruption of *itpk1* has a pronounced effect on inositol—perhaps implying that the pleiotropic effects of ITPK1 on plant physiology, including those attributed to perturbation of inositol pyrophosphate signaling, may have their origins more broadly in inositol phosphate metabolism. An elevation of inositol was observed in *itpk2* down-regulated rice RNAi [23,24]. These data potentially explain the reductions in labeling of InsP_6_ from exogenous [^3^H]-inositol observed in vegetative tissues of *itpk1* [27], by simple dilution of label. An alternative mechanism for the elevation of inositol levels in *itpk1* may lie in the equivalence of the roles of plant ITPK1 and mammalian inositol hexakisphosphate kinase (IP6K)1 for the phosphorylation of InsP_6_ [28–32]: Greenberg et al. [49] showed that the disruption of IP6K1 (loss of 5-PP-InsP_5_) increases MIPS (ISYN, mINO1) transcription and elevates inositol levels.

## Methods

### Protein expression and purification

### *Af*IPS (MIPS)

*Archaeoglobus fulgidus* inositol phosphate synthase (AfIPS, MIPS), pET23a: *Af*IPS (from Adolfo Saiardi), was expressed in Rosetta^TM^ 2 (DE3)pLysS (Novagen). Cells were induced at 30°C with 0.5 M isopropyl β-D-1-thiogalactopyranoside overnight in LB medium with ampicillin and chloramphenicol selection. Cells were resuspended in lysis buffer and lysed by French press in 50 mM NaH_2_PO_4_ pH 7.5, 300 mM NaCl, 20 mM imidazole, 1% Triton-X-100, and protease inhibitor (Roche). It was determined that the protein did not interact with the nickel-nitriloacetic acid (NiNTA) column since the NiNTA loading waste and wash A (50 mM NaH_2_PO_4_ pH 7.5, 300 mM NaCl, 20 mM imidazole) contained the target protein, but wash B (50 mM NaH_2_PO_4_ pH 7.5, 300 mM NaCl, 250 mM imidazole) did not (Supplementary Figure S2). Lysate containing 2 mM DTT was heated to 80°C for 30 min and centrifuged at 14,000 x *g* for 15 min. Protein was concentrated to approximately 2 mg/mL and stored at −80°C. Enzyme activity was verified using the assay described below.

*At*ITPK1 and *At*IPK1 were purified as described [28].

### Enzyme assays

### Three-step synthesis of [^32^P]-Ins3P

Conversion of glucose to Ins3P was carried out as a three-step process. First, glucose was converted to glucose-6-phosphate (G6P) in 50 mM Tris-Ac pH 7.5 buffer containing 50 mM D-glucose, 1.5 mM ATP, 1850 kBq γ-^32^P ATP, 5 mM phosphocreatine, 5 mM MgCl_2_, 0.4 units yeast hexokinase, and 6 units of creatine phosphokinase (from rabbit muscle, Merck Product # C3755) in 40 μL at 30°C for 2 h. To ensure all glucose was converted to G6P, further regeneration assay components were added (50 mM phosphocreatine, 15 units creatine phosphokinase, 1 mM DTT, 100 mM NaCl, 3 mM MgCl_2_) and incubated at room temperature for a further 3 h. Finally, to convert G6P to Ins3P, 1 mM NAD^+^, 1 mM ZnSO_4_, and 20 μg IPS enzyme were added to the previous reaction and incubated at 80°C for 4 h.

## ITPK1 assays

Assays without radioactivity were carried out under regeneration conditions as described [28] in 10 μl reactions containing 20 mM HEPES pH 7.5, 1 mM MgCl_2_ at either 100 μM substrate, 200 μM ATP or 500 μM substrate, 500 μM ATP with 1.26 μM *At*ITPK1. Reactions were either incubated overnight or as specified in the text.

Assays with [^32^P]-Ins3P substrate were performed under regeneration conditions, with 2.5 mM substrate, 1 mM ATP, 15 mM phosphocreatine with 1.26 μM protein(s) for 30 min, 1 h, 2 h, 4 h, or 6 h. An aliquot (30 μL) of a hydrolysate of InsP_6_ was added immediately prior to HPLC injection, with the sample made up to 50 μL with water.

Assays with Ins3P and [^32^P]-ATP substrate were performed under regeneration conditions, with 2.5 mM substrate, 1 mM ATP, 1850 kBq [γ-^32^P]-ATP, 15 mM phosphocreatine with 1.26 μM protein(s) for 30 min, 1 h, 4 h, 8 h, or 24 h. An aliquot (30 μL) of a hydrolysate of InsP_6_ was added immediately prior to HPLC injection, with the sample made up to 50 μL with water.

Reactions were stopped by the addition of an equal volume of 60 mM (NH_4_)_2_HPO_4_, pH 3.35, or, for suppressed ion-conductivity analysis, by the addition of 3 volumes of 20 mM disodium EDTA, 100 mM NaF. Products were centrifuged at 14,000 x *g* for 5 min, and transferred to autosampler vials with approximately 50–70% of the reaction products injected.

### Inositol phosphates

Compounds were obtained from Cayman Chemicals or from other sources described in [28,29,50]. Synthetic inositol phosphates were purified by ion-exchange chromatography, eluting with a gradient of triethylammonium bicarbonate, and were fully characterized as their triethylammonium salts by ^1^H and ^31^P NMR spectroscopy and MS, and were shown by HPLC to be >95% pure.

### HPLC of inositol phosphates

### CarboPac PA200

Products of enzyme assays were resolved by anion exchange on CarboPac PA200 (Dionex) eluted with methanesulfonic acid or HCl, and were detected by complexation with ferric ion [28]. For some assays, the gradient of methanesulfonic acid employed was changed: time (min), % B (0.6 M MeSA); 0,0; 25,25; 100,38; 45,100.

### AS11

To compare rates of phosphorylation of Ins1P and Ins3P, assay products were also analyzed by suppressed-ion conductivity on a Dionex (UK) ICS-2100 system after resolution on a 250 × 2 mm AS11 (Dionex) column with a 50 × 2 mm AG11 (Dionex) guard column eluted at a flow rate of 0.35 mL min^-1^ with KOH. The gradient: time (min), KOH (mM); 0,5; 40, 80; was delivered with gradient function ‘5’ in the Chromeleon v.6 software (Dionex, UK) with the anion suppressor current set at 99 mA and the column oven at 30°C. The column was washed with 5 mM KOH for 10 min between injections. Ins1P and Ins3P stocks (Cayman Chemicals) were initially analyzed on a gradient: time (min), KOH (mM); 0,5; 80,80; to normalize concentrations before inclusion at 500 μM in the enzyme assay. While the injected sample contained components that obscure the substrate peak and that of potential InsP_2_ and InsP_3_ products, viz. buffer components and ‘stopping’ reagents, baseline resolution of phosphate, ATP, and InsP_4_ and Ins(1,3,4,5,6)P_5_ products was obtained. The absence of appreciable accumulation of InsP_2_ and InsP_3_ products was verified in the same samples by post-column detection with ferric nitrate for which the ‘stopping’ reagents do not interfere.

The Ins(1,3,4,5,6)P_5_ product peak (of Figure 7) was integrated with Chromeleon software, referenced to Ins(1,3,4,5,6)P_5_ standard. The rate and standard error of the measurement from single determinations at each time point was obtained from the regression line of the time course, taking account of protein content of the assay.

### Partisphere SAX

Radiometric assays were analyzed by on-line Cerenkov counting in either a Radiomatic A515 series (Canberra Packard, UK) or a β-Ram 5 (LabLogic, UK) flow detector, both fitted with a 500 μL flow cell, using an integration interval of 1 s. For these assays, reaction products were resolved on a 250 × 4.6 mm Partisphere SAX (Whatman, obtained from Hichrom, UK) column maintained at 30°C and eluted at 0.8 mL min^-1^. The gradient, made from water [A] and 1.25 M (NH_4_)_2_HPO_4_, pH 3.8 adjusted with H_3_PO_4_ [B], was constructed: time (min), % B; 0,0; 5,0; 65,100; 75,100. A UV detector placed upstream of the radio detector was set at 254 nm to monitor elution of nucleotide; the signal also reports development of the gradient. Peaks were integrated with the Flo-One for Windows software of the flow detector, with the Ins(1,3,4,5,6)P_5_ peak (of Figure 5) expressed as a % of counts recovered in the summed peaks of the gradient. The standard error of the linear regression as calculated by GraphPad Prism v.6 yielded a rate constant of 19.42 ± 0.800 nmol min^-1^ mg^-1^ (0.70 ± 0.029 min^-1^), from single determinations at each time point. The calculation takes account of the protein content of the assay and starting substrate concentration.

All HPLC data were exported as ASCII files and re-plotted in GraphPad Prism v6.0 without data smoothing or manipulation, other than offset on the *y*-scale of individual figures to aid clarity.

### Measurement of inositol

Inositol was separated from sugars by 2D-HPLC on CarboPac PA1 (Dionex) and MA1 (Dionex) columns with detection by pulsed amperometry on the gold electrode of a Dionex DX600 HPLC machine [51]. For this, inositol was extracted by grinding of seeds in a liquid nitrogen-chilled 1.5 mL Eppendorf tube using a polypropylene micro-pestle with subsequent extraction for 30 min on ice in 300 μL 0.6 M HCl, with further grinding. Samples were diluted 10–20x in 18.2 MOhm cm water before injection of 20 μL aliquots. Peaks were integrated in Chromeleon software and quantified against a calibration curve of 10–200 pmol injections in 20 μL.

### Genotypes

Genotypes employed in this study were described [27] or were obtained by genetic crosses of genotypes described therein.

## Supplementary material

online supplementary material 1.

## Data Availability

The authors affirm that all data supporting the findings of the study are available within the article and supplementary materials.

## Open Access

Open access for this article was enabled by the participation of the University of East Anglia in an all-inclusive Read & Publish agreement with Portland Press and the Biochemical Society under a transformative agreement with JISC.

## Abbreviations

*Af*IPS: *Archeoglobus fulgidus* inositol phosphate synthase
*At*IPK1: *Arabidopsis thaliana* inositol pentakisphosphate 2-kinase
*At*ITPK1: *Arabidopsis thaliana* inositol tris/tetrakisphosphate kinase 1
DTT: reduced dithiothreitol
G6P: glucose-6-phosphate
IPK1: inositol pentakisphosphate 2-kinase
IP6K: inositol hexakisphosphate kinase
IPMK/IPK2: inositol polyphosphate multikinase
ITPK: inositol tris/tetrakisphosphate
Ins1P: 1D-*myo*-inositol 1-monophosphate
Ins3P: 1D-*myo*-inositol 3-monophosphate
Ins4P: 1D-*myo*-inositol 4-monophosphate
Ins(1,3)P_2_: *myo*(meso)-inositol 1,3-bisphosphate
Ins(1,4)P_2_: 1D-*myo*-inositol 1,4-bisphosphate
Ins(3,4)P_2_: 1D-*myo*-inositol 3,4-bisphosphate
Ins(4,5)P_2_: 1D-*myo*-inositol 4,5-bisphosphate
Ins(1,4,5)P_3_: 1D-*myo*-inositol 1,4,5-trisphosphate
Ins(3,4,5)P_3_: 1D-*myo*-inositol 3,4,5-trisphosphate
Ins(3,4,6)P_3_: 1D-*myo*-inositol 3,4,6-trisphosphate
Ins(1,3,4,5)P_4_: 1D-*myo*-inositol 1,3,4,5-tetrakisphosphate
Ins(1,3,4,6)P_4_: *myo*-inositol 1,3,4,6-tetrakisphosphate
Ins(1,3,5,6)P_4_: 1D-*myo*-inositol 1,3,5,6-tetrakisphosphate
Ins(1,4,5,6)P_4_: 1D-*myo*-inositol 1,4,5,6-tetrakisphosphate
Ins(3,4,5,6)P_4_: 1D-*myo*-inositol 3,4,5,6-tetrakisphosphate
Ins(1,3,4,5,6)P_5_: *myo*-inositol 1,3,4,5,6-pentakisphosphate
InsP_6_/Ins(1,2,3,4,5,6)P_6_: *myo*-inositol 1,2,3,4,5,6-hexakisphosphate
MIPS: D-*myo*-inositol 3-phosphate synthase
NiNTA: nickel-nitrilotriacetic acid
1-PP-InsP_5_: 1-diphosphoinositol pentakisphosphate
3-PP-InsP_5_: 3-diphosphoinositol pentakisphosphate
5-PP-InsP_5_: 5-diphosphoinositol 1,2,3,4,6-pentakisphosphate
1,5-(PP)_2_-InsP_4_: bis-1,5-diphospho-myo-inositol tetrakisphosphate
3,5-(PP)_2_-InsP_4_: bis-3,5-diphospho-myo-inositol tetrakisphosphate
Ptd: Phosphatidyl
*St*ITPK: *Solanum tuberosum* inositol tris/tetrakisphosphate kinase
VIH: *Arabidopsis thaliana* diphosphoinositol pentakisphosphate kinase
WT: wildtype
