# Supplementary material for: A minimum catalytic unit for synthesis of InsP6 and 5-PP-InsP5 in Arabidopsis
Source: Biochem J. 2025 Dec 17;482(24):1815–30. doi: 10.1042/BCJ20253161 (PMC7618535; doi:10.1042/BCJ20253161)
Supplement: online supplementary material 1. [file bcj-482-24-BCJ20253161-s001.docx]

Supplementary Information

A minimum catalytic unit for synthesis of InsP_6_ and 5-PP-InsP_5_ in Arabidopsis

Hayley L. Whitfield^1^, Colleen Sprigg^1^, Andrew M. Riley^2^, Barry V.L. Potter^2^, Hui-Fen Kuo^3^, Charles A. Brearley^1^

^1^School of Biological Sciences, University of East Anglia, Norwich Research Park, Norwich NR4 7TJ, UK

^2^Medicinal Chemistry & Drug Discovery, Department of Pharmacology, University of Oxford, Oxford OX1 3QT, UK

^3^Agricultural Biotechnology Research Centre, Academia Sinica, Taipei 115, Taiwan

Figure S1 Analysis of Ins1P and Ins3P phosphorylation on CarboPac PA200

Figure S2 SDS-PAGE analysis of *Af*IPS expression


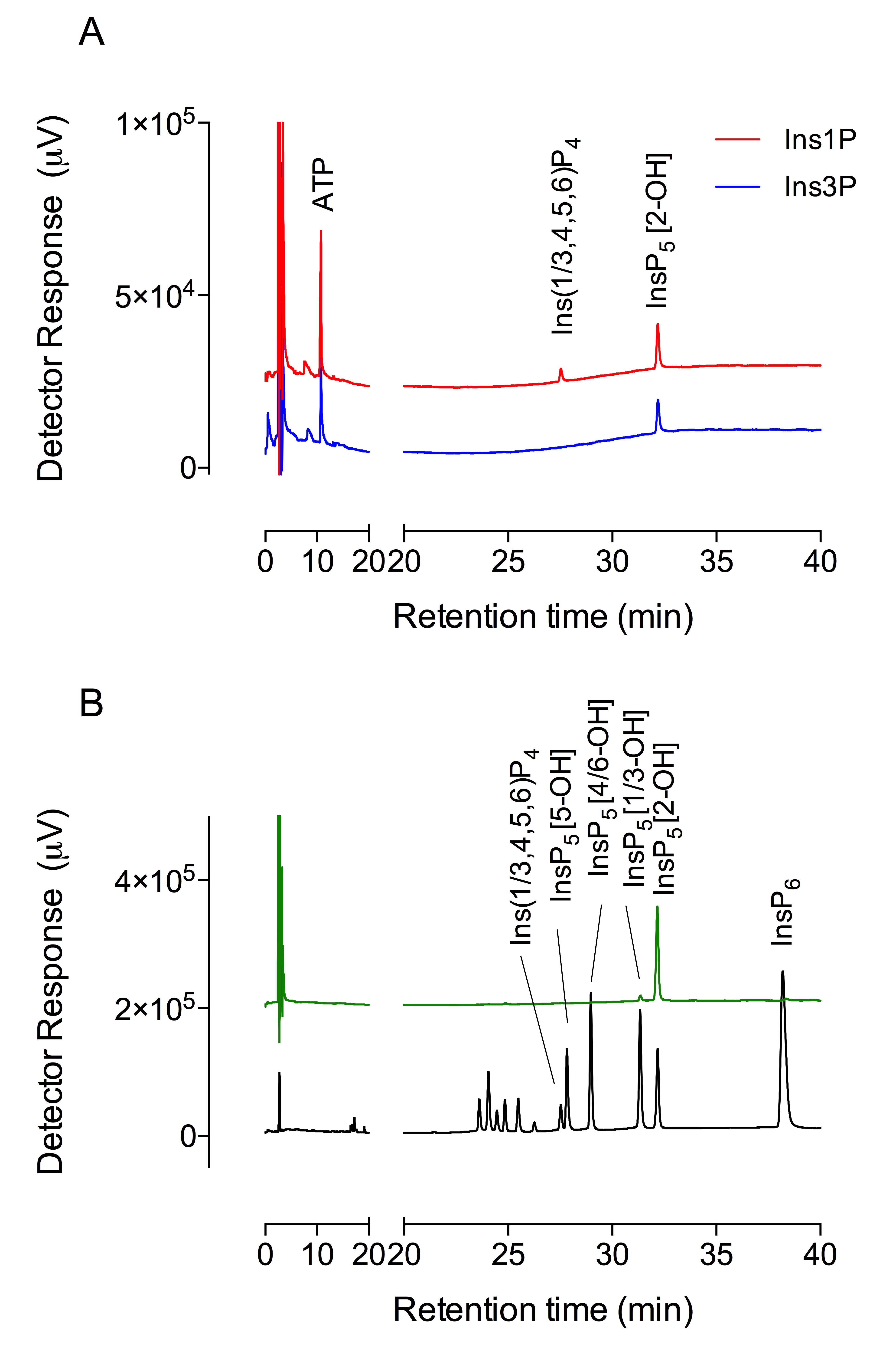


**Figure S1. Analysis of Ins1P and Ins3P phosphorylation**. Assays stopped by the addition of NaF, EDTA, pH 10, were eluted on a CarboPac PA200 column. **A**, products generated from Ins1P (red trace) share retention time with standards: Ins(1/3,4,5,6)P_4_ (identified in an hydrolysate of InsP_6_, black trace in B) and Ins(1,3,4,5,6)P_5_ (InsP_5_ [2-OH], green trace in B). Products generated from Ins3P (blue trace) share retention time with InsP_5_ [2-OH]. The position of elution of ATP is shown. The gradient of methanesulfonic acid used was : time (min), % B (0.6 M MeSA); 0,0; 25,25; 100,38; 45,100. The chromatography shown in the figure has been repeated on more than ten occasions. Ins1P, Ins3P and EDTA elute in the solvent front at c. 2.5-3 minutes.

**
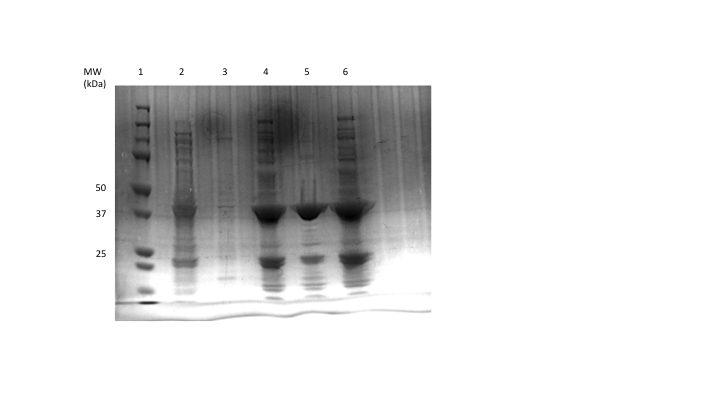
**

**Figure S2. SDS-PAGE of pET23a: *Af*IPS, expressed in Rosetta^TM^ 2 (DE3)pLysS (Novagen).** Lanes (interspersed with unloaded lanes): 1, Ladder; 2, NiNTA wash A (50 mM NaH_2_PO_4_ pH 7.5, 300 mM NaCl, 20 mM imidazole); 3, NiNTA wash B (50 mM NaH_2_PO_4_ pH 7.5, 300 mM NaCl, 250 mM imidazole); 4, 60°C in 2 mM DTT for 30 mins; 5, 80°C in 2 mM DTT for 30 mins; 6, 40°C for 1 hr then 60°C for 30 mins.
